# Exploring the role of differentially expressed metabolic genes and their mechanisms in bone metastatic prostate cancer

**DOI:** 10.7717/peerj.15013

**Published:** 2023-04-12

**Authors:** Qingfu Zhang, Peng Zhang, Zhongting Zhao, Jun Wang, Hepeng Zhang

**Affiliations:** 1 Department of Urology, Tai ’an Central Hospital, Tai ’an, Shandong, China; 2 Department of Spine Surgery, Tai ’an Central Hospital, Tai ’an, Shandong, China; 3 Department of Spinal Surgery, The Third People’s Hospital of Jinan, Jinan, Shandong, China; 4 Department of Emergency, Qingdao Eighth People’s Hospital, Qingdao, China

**Keywords:** Prostate cancer (PCa), Bone metastasis (BM), Epithelial to mesenchymal transition (EMT), Immuno-microenvironmental homeostasis, scRNA-seq

## Abstract

**Background:**

Approximately 10–20% of patients diagnosed with prostate cancer (PCa) evolve into castration-resistant prostate cancer (CRPC), while nearly 90% of patients with metastatic CRPC (mCRPC) exhibit osseous metastases (BM). These BM are intimately correlated with the stability of the tumour microenvironment.

**Purpose:**

This study aspires to uncover the metabolism-related genes and the underlying mechanisms responsible for bone metastatic prostate cancer (BMPCa).

**Methods:**

Gene Expression Omnibus (GEO) and The Cancer Genome Atlas (TCGA) datasets of PCa and BM were analyzed through R Studio software to identify differentially expressed genes (DEGs). The DEGs underwent functional enrichment via Kyoto Encyclopedia of Genes and Genomes (KEGG) and Gene Ontology (GO), with key factors screened by a random forest utilized to establish a prognostic model for PCa. The study explored the relationship between DEGs and the stability of the immune microenvironment. The action and specificity of CRISP3 in PCa was validated through western blot analysis, CCK-8 assay, scratch assay, and cellular assay.

**Results:**

The screening of GEO and TCGA datasets resulted in the identification of 199 co-differential genes. Three DEGs, including DES, HBB, and SLPI, were selected by random forest classification model and cox regression model. Immuno-infiltration analysis disclosed that a higher infiltration of naïve B cells and resting CD4 memory T cells occurred in the high-expression group of DES, whereas infiltration of resting M1 macrophages and NK cells was greater in the low-expression group of DES. A significant infiltration of neutrophils was observed in the high-expression group of HBB, while greater infiltration of gamma delta T cells and M1 macrophages was noted in the low-expression group of HBB. Resting dendritic cells, CD8 T cells, and resting T regulatory cells (Tregs) infiltrated significantly in the high-expression group of SLPI, while only resting mast cells infiltrated significantly in the low-expression group of SLPI. CRISP3 was established as a critical gene in BMPCa linked to DES expression. Targeting CRISP3, d-glucopyranose may impact tumour prognosis. During the mechanistic experiments, it was established that CRISP3 can advance the proliferation and metastatic potential of PCa by advancing epithelial-to-mesenchymal transition (EMT).

**Conclusion:**

By modulating lipid metabolism and maintaining immunological and microenvironmental balance, DES, HBB, and SLPI suppress prostate cancer cell growth. The presence of DES-associated CRISP3 is a harbinger of unfavorable outcomes in prostate cancer and may escalate tumor proliferation and metastatic capabilities by inducing epithelial-mesenchymal transition.

## Introduction

The incidence of prostate cancer (PCa) is 9% worldwide (Siegel et al., 2022), and about 10–20% of PCa patients develop castration-resistant prostate cancer (CRPC) (Kirby, Hirst & Crawford, 2011), of which 90% of the patients with metastatic CRPC (mCRPC) develop bone metastases (BM) (Gandaglia et al., 2015). Bone metastatic prostate cancer (BMPCa) is mainly characterized by osteoblastic lesions with the formation of osteolytic components (Roudier et al., 2004). PCa cells usually lead to the activation of bone anabolic pathways and reduced bone deposition quality, so that the spatial distribution of the bone matrix is disturbed even in the presence of active osteoblasts, causing microstructural damage and reduced mechanical resistance in the bones (Sekita, Matsugaki & Nakano, 2017; Wong et al., 2019). Therefore, patients with BM are often at an increased risk of fractures, bone pain, and disability, with a poor prognosis (Bubendorf et al., 2000). In order to improve the prognosis of patients with PCa, it is important to study BM formed from PCa.

Preclinical models of BM suggest that tumors can influence the bone marrow microenvironment at an early stage of the metastatic process by forming pre-metastatic niches (Giles et al., 2016; Peinado, Lavotshkin & Lyden, 2011). After colonization, tumor cell survival and clonal selection are dependent on the bone microenvironment, which includes angiogenesis and reprogramming of stromal signaling, immune regulation, and immune escape (Hofbauer et al., 2021). In the bone microenvironment, different types of immune cells exert different tumor-specific effects on the formation and progression of BM. For example, the expression of the macrophage marker, CD68, is upregulated in BMPCa, and the number of phagocytic CD68 ^+^ cells in PCa patients is positively correlated with their Gleason score (Rusthoven et al., 2014; Gucalp et al., 2017). Moreover, most cancer-related deaths occur due to metastasis, which is governed by the tumour microenvironment (Chambers, Groom & MacDonald, 2002; Lambert, Pattabiraman & Weinberg, 2017). Therefore, an in-depth understanding of the micro-environment immune mechanisms will contribute to the prevention and treatment of metastatic tumors.

Currently, paclitaxel is primarily used for the treatment of BMPCa; however, the majority of mCRPC patients eventually develop resistance against paclitaxel (Body, Casimiro & Costa, 2015). Therefore, elucidating the molecules associated with the development of BM is necessary to improve the prognosis of BMPCa patients. Effective treatment strategies are still lacking for patients with drug-resistant BM. With computerised genetic technology, fields like medicine and healthcare will be able to provide reliable support for curing entire populations with the help of bioinformatics (Wooller et al., 2017; Kinghorn et al., 2017; Sun et al., 2018; Li et al., 2019; Shi et al., 2021; Chen et al., 2021; Chen et al., 2022; Xuan et al., 2022; Lin et al., 2022). This study aims to explore the metabolism-related genes and the underlying mechanisms associated with BMPCa, using bioinformatics analysis.

## Methods

### Differential gene analysis

The data were downloaded from The Cancer Genome Atlas (TCGA) database (https://portal.gdc.cancer.gov/) and contained 177 normal and five PCa samples. DEGs were identified using the R software package DEGseq2, *p*-value <0.05, and log2FC (—log2FC—) >1. To perform external validation, GEO datasets (https://www.ncbi.nlm.nih.gov/geo/) were filtered by entering “metastatic” and “prostate cancer” in the search box. The following inclusion criteria must be met for data to be included in the database: the data information must be detailed and downloadable and; the sample size must be sufficient. Datasets for GSE32269 were downloaded from the GEO database (https://www.ncbi.nlm.nih.gov/geo/) (Cai et al., 2013). In this data set, 51 PCa and four BMPCa samples (*n* = 55) were annotated using the GPL96 platform.

### Quality control and analysis of single cell RNA (scRNA)-seq data

To retrieve relevant data, “single-cell RNA sequencing” and “prostate cancer” were entered into the NCBI GEO database (http://www.ncbi.nlm.nih.gov/geo/). The study was conducted using the GSE168733 dataset obtained from the Gene Expression Omnibus (GEO) database, containing information from three cell types, including LNCaP, RES-A, and RES-B cells (*n* = 3000 cells) (Taavitsainen et al., 2021). We used the avereps function of the limma package in R SOFTWARE 4.1.2 to obtain the average values of the data. The scRNA-seq data was subjected to quality control, after which it was statistically analyzed using the Seurat package. Samples with a minimum cell count of <3 and cells with <50 features were first filtered out, and then cells with ≥20% of mitochondria expressing genes were excluded. A total of 143 low quality cells were excluded and only data from 2,857 cells were included in the analysis. The data were log-normalized (log[10,000UMI gene /UMI cell +1]). The top 1,500 genes between cells were extracted and subjected to principle component analysis (PCA). After normalizing the data, the t-distributed random neighborhood embedding (tSNE) algorithm was used to reduce the dimensionality of the 20 initial principle components (PC) and cluster analysis was performed to obtain different clusters. Wilcox was used to find differential genes for each cluster, and heat maps were drawn based on the top 10 genes for each cluster. Different cell clusters were identified and annotated using the singleR package, which was then manually validated and corrected (Taavitsainen et al., 2021).

### Functional enrichment analysis

For functional enrichment analysis of the DEGs, we first converted the gene name into Entrez ID using the R package (org.Hs.eg.db; https://bioconductor.org/packages/release/data/annotation/html/org.Hs.eg.db.html). The DEGs were then subjected to enrichment analysis using KEGG and GO and scored using the following formula: Enrichment score = (overlapGeneCount × bgGeneNum)/(diffGeneNum × termGeneNum). The results were visualized using the R packages clusterProfiler and ggplot2.

### Random forest screening of key factors

Perl language and machine learning random forest algorithm were used to extract approximately 199 genes from the intersection of the TCGA dataset. The decision tree size was 500 after data centering. Genes were screened based on cross-validation of the data.

### Prognostic model construction

Prognostic analysis was performed on the DEGs, and the expression data and clinical information of the genes were obtained from the TCGA database. Cox regression analysis was performed on the DEGs using R software, survival package, and survminer package to determine the prognostic genes. The genes with AUC >0.6 were identified by receiver operating characteristic (ROC) validation, and Kaplan–Meier (KM) survival curves were constructed for the three genes (*DES*, *HBB*, and *SLPI*), with the best cutoff value for each gene derived using surv_cutpoint function.

### High and low gene expression grouping and enrichment analysis

The PCa samples were categorized into high- or low-expression groups based on to the median log2 expression levels of *DES*, *HBB*, and *SLPI*, respectively. Differential analysis was performed for each group using limma package, and heatmap visualization of the 10 most significantly expressed DEGs was done by using the ggplot2 package. Each gene was analyzed using gene set variation analysis (GSVA) package to identify the associated pathways.

### Monogenic immune infiltration

CIBERSORT analysis was performed to determine the variations in the immune infiltration of 22 immune cells between the high- and low-*DES*, *HBB*, and *SLPI* subgroups, and the results were analyzed by two independent samples rank sum test as previous researches (Newman et al., 2015; Chen et al., 2018; Kang et al., 2021; Kawada et al., 2021; Deng et al., 2021; Mei, Li & Kang, 2022). Thereafter, correlation analysis was conducted for the 22 immune cells and the three genes by using spearman correlation analysis.

### Cell culture

From Procell (Wuhan, China), we procured a collection of human prostate cancer cell lines (PC3, DU145, LNCaP, and 22RV1). The cells were incubated in RPMI-1640 (bl303a; Biosharp, Anhui, China) at 37 °C under an atmosphere of 5% CO2. The cells were firmly affixed to the culture vessel and subcultured every 72 h.

### Transfection and grouping

Employing logarithmically growing LNCaP cells (*n* = 200,000), Lipofectamine 2000 (11668-027; Invitrogen, Waltham, MA, USA) was introduced *via* transfection in accordance with the manufacturer’s protocol. Three groups were evaluated for their CRISP3 expression: low expression of CRISP3 (si-CRISP3), low expression with CRISP3 overexpression (over-CRISP3), and the absence of CRISP3 expression. The efficacy of the transfection was verified through quantitative reverse transcription-polymerase chain reaction (qRT-PCR). The experiment was conducted thrice to establish reproducibility. The primer sequences, as previously reported, are displayed in Table S1 (Wang et al., 2022).

### RNA isolation and qRT-PCR

Total RNA was extracted from LNCaP cells with TRIzol reagent (Invitrogen, Carlsbad, CA, USA). The detection of the total RNA was performed using HiScript III RT SuperMix (Vazyme, Nanjing, China) and reverse transcription was carried out with HiScript III RT SuperMix (Vazyme, Nanjing, China). Glyceraldehyde 3-phosphate dehydrogenase (GAPDH) was utilized as an internal standard. The primer sequences, as previously reported, are displayed in Table S2 (Wang et al., 2022).

### Western blot analysis

Lysates of protein samples obtained from tissues or cells *via* RIPA buffer were subjected to 10% sodium dodecyl sulfate-polyacrylamide gel electrophoresis (SDS-PAGE) and then transferred to polyvinylidene fluoride membranes. The antibodies used included anti- *β*-catenin, anti-Vimentin, and anti-Snail, while *β*-Tubulin served as the internal control.

### CCK-8 assay

The logarithmically growing LNCaP cells were seeded into culture plates, as previously documented (Yang et al., 2022). The experimental groups underwent the corresponding treatments before 10 µL of CCK-8 solution (PR645; Dojindo, Kumamoto, Japan) was added per well. The culture plates were then incubated in SpectraMax i3 microplate readers (Molecular Devices, San Jose, CA, USA). The rate of proliferation inhibition was calculated as follows: (control absorbance value - experimental absorbance value)/control absorbance value ×100%. The experiment was conducted thrice.

### Scratch assay

The cells were seeded into 6-well plates and underwent grouping and transfection after reaching 80% confluence. After 24 h of scratching with a sterile gun tip, the cells were supplemented with RPMI-1640 culture medium containing 0.5% serum. The images were analyzed using ImageJ software.

### Statistical analysis

Continuous variables were analyzed using the Student’s *t*-test and categorical variables were analyzed using the chi-squared test. Means and standard deviations were calculated, and data analysis was performed using R version 4.1.2 and GraphPad Prism 8.0 software. Differences were considered significant at *p*-values less than 0.05. The experiment was conducted thrice.

## Results

### Screening for BMPC-associated DEGs

**Figure 1:**
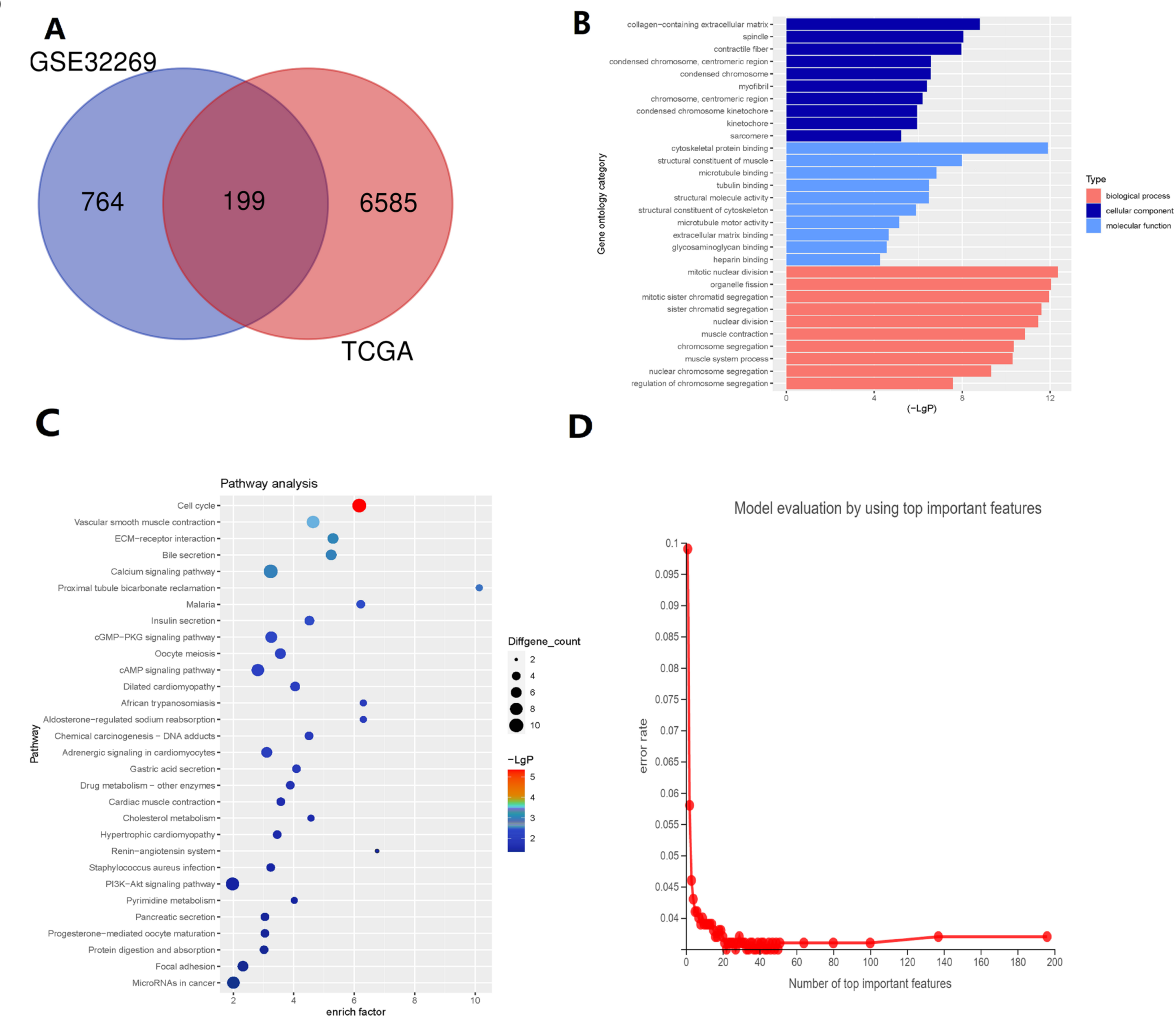
Identification of key genes *via* TCGA and GSE32269 Datasets. (A) Intersecting the differential genes of the TCGA and GSE32269 datasets, yielding 199 common genes. (B) Bar graphs depicting the Gene Ontology enrichment analysis of the differential genes. (C) Bubble plots portraying the Kyoto Encyclopedia of Genes and Genomes enrichment analysis of the differential genes. (D) Fold plots displaying error rates and the premier genes.

There are 199 intersecting DEGs between TCGA and GSE32269 (Fig. 1A). GO functions with the highest scores in each item are displayed in the enrichment analysis chart (Fig. 1B). KEGG enrichment analysis was used to construct the distribution map of the significant differential gene pathways based on their significance level and the number of DEGs included in the analysis (*p* value <0.05) (Fig. 1C). As shown in the Fig. 1C, cell cycle and vascular smooth muscle contraction have the highest scores. In these results, pathways and functions are identified in which these 199 DEGs are primarily enriched.

**Figure 2:**
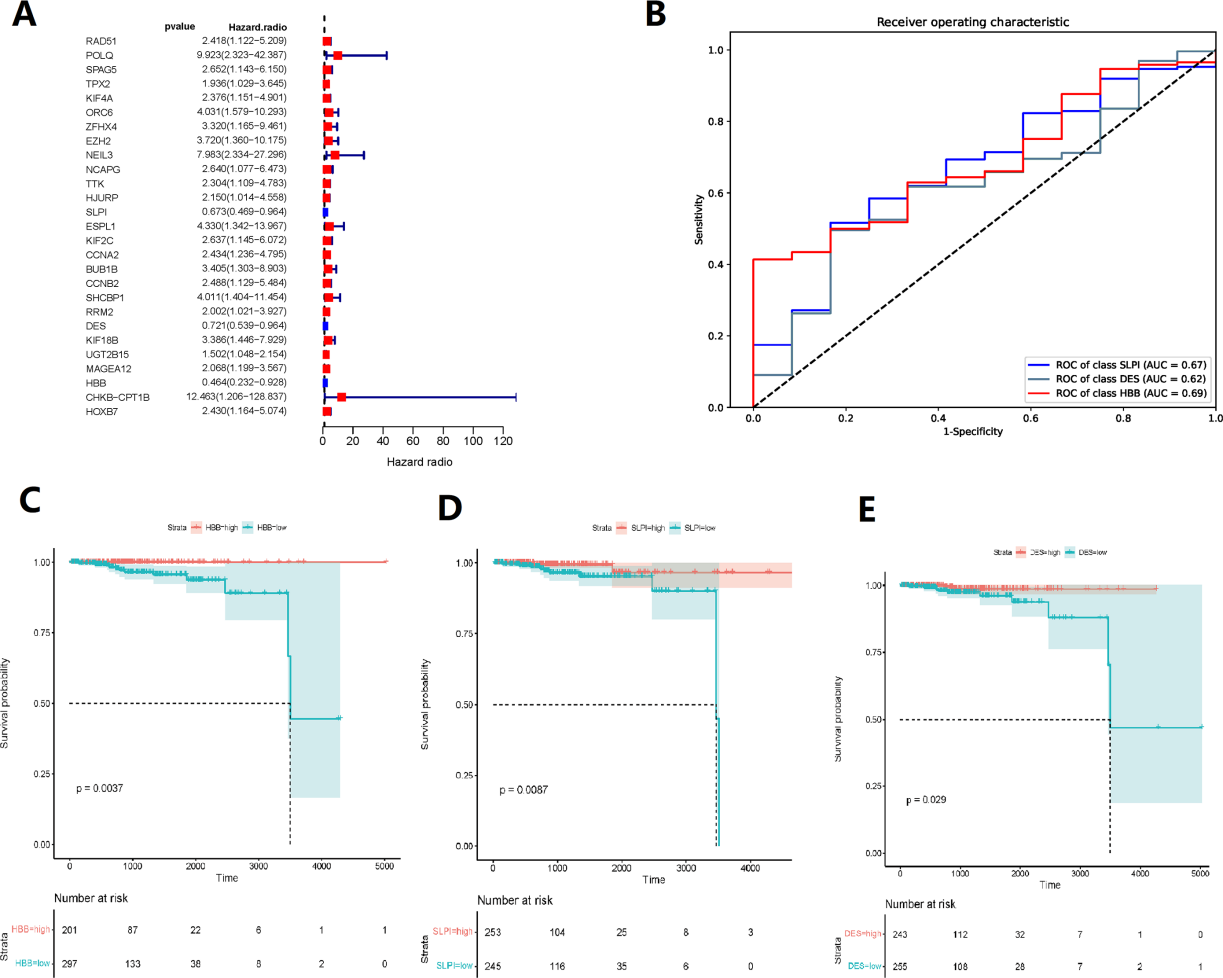
TCGA data-based cox regression analysis. (A) Identification of 27 genes relevant to prognosis of bone metastatic prostate cancer (BMPCa); (B) selection of Genes with AOC greater than 0.6; (C) Kaplan–Meier Survival Curves for HBB, (D) SLPI, and (E) DES.

Random forest classification model was used to score the DEGs based on their importance (Fig. 1D). In order to demonstrate the effectiveness of the random forest model, we further demonstrated the top 20 DEGs (Fig. S1). The prognostic model was constructed using the results of the random forest model, leading to 27 BMPCa prognostic genes. and the results were validated using ROC curves to obtain three genes, including desminopathies (*DES*), beta-globin gene (*HBB*), and secretory leukocyte protease inhibitor (*SLPI*) (Figs. 2A–2B). KM survival curves with low expression for *HBB*, *SLPI*, and *DES* indicate poor tumour outcomes (Figs. 2D–2E). And *HBB*, *SLPI,* and *DES* may be BMPC-related DEGs.

### Differential gene and enrichment analysis related to *HBB*, *SLPI*, and *DES*

**Figure 3:**
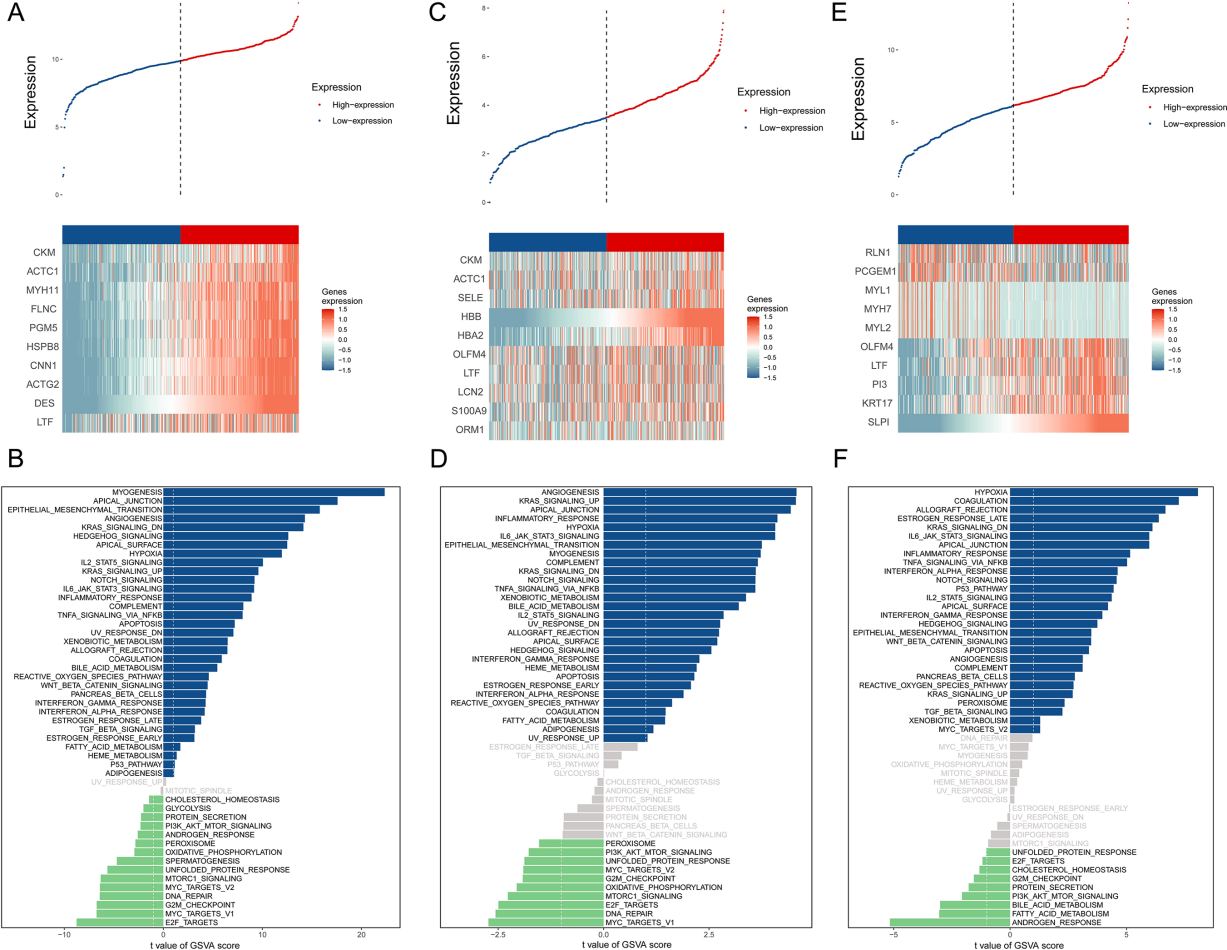
An assessment of DES, HBB, and SLPI using TCGA Data. (A) Comparison of High- and Low-DES gene expressions through heat map analysis; (B) analysis of pathway variations between high- and low-DES expression groups through GSVA; (C) comparison of high- and low-HBB gene expressions through heat map analysis; (D) analysis of pathway enrichments between high- and low-HBB Expression groups using GSVA; (E) comparison of high- and low-SLPI gene expressions through heat map analysis; (F) analysis of pathways linked to high- and low-SLPI expressions.

The BMPCa samples were grouped into high- and low-expression groups according to the expression levels of *DES*, *HBB*, and *SLPI*. Cdk8-dependent kinase module (*CKM*), alpha cardiac muscle 1 (*ACTC1*), myosin heavy chain 11 (*MYH11*), filamin C (*FLNC*), phosphoglucomutase 5 (*PGM5*), heat shock protein B8 (*HSPB8*), calponin 1 (*CNN1*), actin gamma 2 (*ACTG2*), and lactotransferrin (*LTF*) showed high expression in the *DES* high-expression group, which was significantly enriched in the pathways associated with myogenesis, apical junction, epithelial mesenchymal transition, and angiogenic transition (Figs. 3A–3B).

*CKM*, *ACTC1*, E-selectin (*SELE*), hemoglobin A2 (*HBA2*), olfactomedin 4 (*OLFM4*), lactoferrin (*LTF*), lipocalin 2 (*LCN2*), *S100A9*, and orosomucoid (*ORM1*) were highly expressed in the *HBB* high-expression group, which was significantly enriched in pathways associated with angiogenesis, KARS signaling pathway, apical junction, and inflammatory response (Figs. 3C–3D).

Perilipin-1 (*PLN1)*, prostate cancer gene expression marker 1 (*PCGEM1*), myosin light chain 1 (*MYL1*), myosin heavy chain 7 (*MYH7*), myosin light chain 2(*MYL2*), olfactomedin 4 (*OLFM4*), lactotransferrin (*LTF*), phosphatidylinositol 3 (*PI3*), and recombinant keratin 17 (*KRT17*) were highly expressed in the *SLPI* high-expression group, which was significantly enriched in pathways associated with hypoxia, coagulation, allograft rejection, and late estrogen response (Figs. 3E–3F).

According to the above studies, *HBB*, *SLPI*, and *DES* are closely related to tumor-associated pathways and microenvironments.

### Monogenic immune infiltration

**Figure 4:**
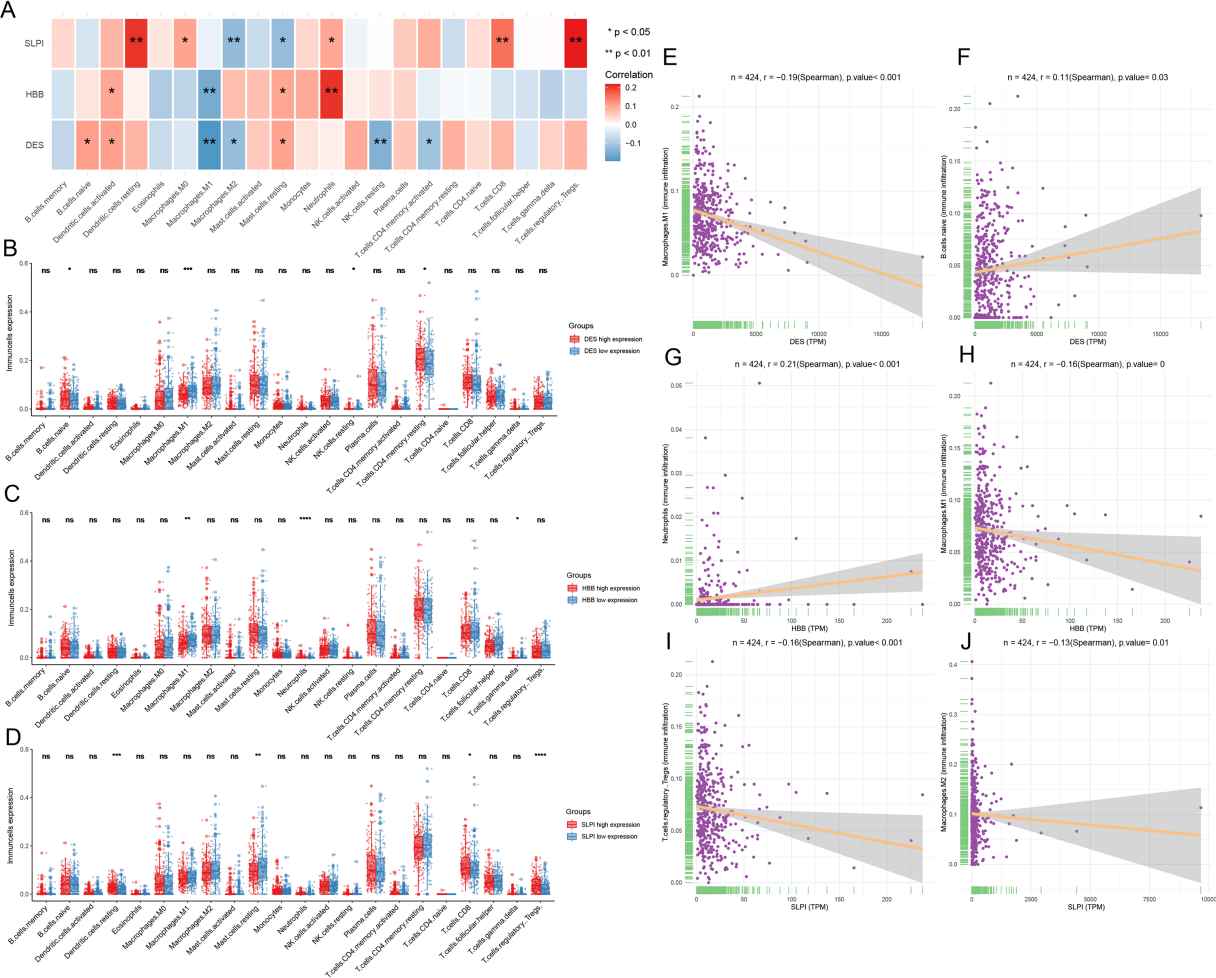
Analysis of immune infiltration by CIBERSORT. (A) assessment of the presence of 22 distinct immune cell types in groups with low or high expression of DES, HBB, and SLPI, respectively; (B) correlation between the expression levels of DES, HBB, and SLPI, and the 22 immune cell types; (C) linear correlation between DES and M1 macrophages; (D) linear correlation between DES and naive B cells; (E) linear correlation between HBB and neutrophils; (F) linear correlation between HBB and M1 macrophages; (G) linear correlation between SLPI and T-regulatory cells; and (H) linear correlation between SLPI and M2 macrophages.

Infiltration of the naïve B cells and resting CD4 memory T cells was greater in the *DES* high-expression group, while M1 macrophage and resting NK cell infiltration was greater in the *DES* low-expression group (Fig. 4A). This suggests that high *DES* expression may promote the infiltration of naïve B cells and resting CD4 memory T cells, which exert anti-tumor effects. Neutrophils were significantly infiltrated in the *HBB* high-expression group, while gamma delta T cell and M1 macrophage infiltration was increased in the *HBB* low-expression group (Fig. 4B). These results suggest that high *HBB* expression may promote neutrophils infiltration. Resting dendritic cell, CD8 T cell, and Treg infiltration was increased in the *SLPI* high-expression group, while resting mast cell infiltration was increased in the *SLPI* low-expression group (Fig. 4C). These results suggests that high *SLPI* expression may promote infiltration of resting dendritic cells, CD8 T cells, and Tregs, which in turn exert anti-tumor effects. Therefore, anti-tumor M1 macrophage infiltration was significantly increased in the *DES* and *HBB* low-expression groups, suggesting that there may be other factors contributing to the poor prognosis of the low-*DES* and *HBB* expressing patients.

Correlation analysis revealed that *DES* expression showed a significant negative correlation with M1 macrophages and a positive correlation with naïve B cells. In contrast, *HBB* expression showed a significant positive correlation with neutrophils and a negative correlation with M1 macrophages. Lastly, *SLPI* expression showed a significant positive correlation with resting dendritic cells, CD8 T cells, and Tregs and a negative correlation with M2 macrophages (Figs. 4E–4J). We suggest that high expression of *SLPI* may inhibit the infiltration of M2 macrophages, thereby suppressing tumor growth.

### Analysis of single cell heterogeneity and marker genes

**Figure 5:**
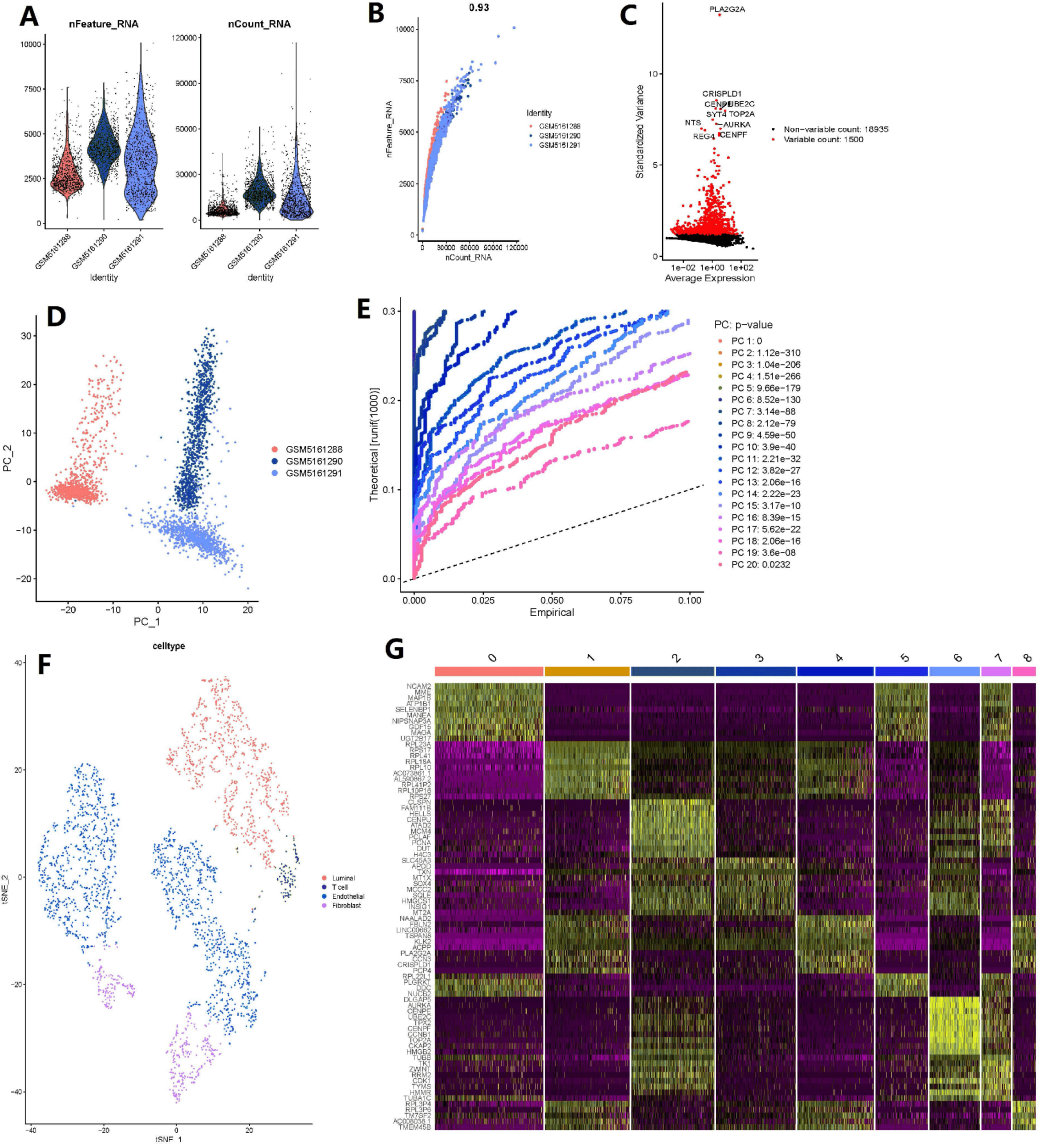
Based on single-cell RNA-seq data, four clusters of prostate cancer (PCa) cells were identified with different annotations. (A) After quality control of 3,000 cells from the tumor cores of three human LNCaP and VCaP samples, 2,857 cells were included in the analysis; (B) the number of genes detected was significantly correlated with the depth of sequencing (Pearson correlation coefficient = 0.93); (C) analysis of variance (ANOVA) plot showing 19,752 corresponding genes in all glioblastoma multiforme cells (GBMs); (D) principle component analysis (PCA) showing clear separation of cells; (E) PCA identified 20 cases of principle component, with estimated *P*-value of <0.05; (F) the 20 pc were downscaled using the t-distributed random neighborhood embedding (tSNE) algorithm, followed by cell type annotation and classification of four cell clusters; and (G) differential analysis identified 8,025 marker genes, and the top 20 marker genes for each cell cluster are shown in the heat map.

The number of genes detected according to the single cell quality control was >0.93, suggesting that they are highly correlated (Figs. 5A–5B). A total of 20,435 corresponding genes were included in the analysis, and analysis of variance (ANOVA) revealed 1500 highly variable genes (Fig. 5C). PCA was used to determine the available dimensions and to screen for correlated genes. Additionally, PCA analysis revealed a significant separation of cells in the three groups (including: GSM5161288, GSM5161290 and GSM5161291) (Fig. 5D). We selected 20 principal components (PCs) with estimated *p*-values <0.05 for further analysis (Fig. 5E). Subsequently, human glioblastoma multiforme cells (GBMs) were divided into nine separate clusters by applying the tSNE algorithm. Cell type annotation was performed for pairs, and four cell types were obtained by annotating them with reference to singleR and previous literature (Fig. 5F). Thereafter, the contribution of the original features to the principal components was shown in Fig. 5G. And marker genes representing prostate cancer heterogeneity were identified.

### CRISP3 was found as a key gene in BMPCa associated with *DES* expression

**Figure 6:**
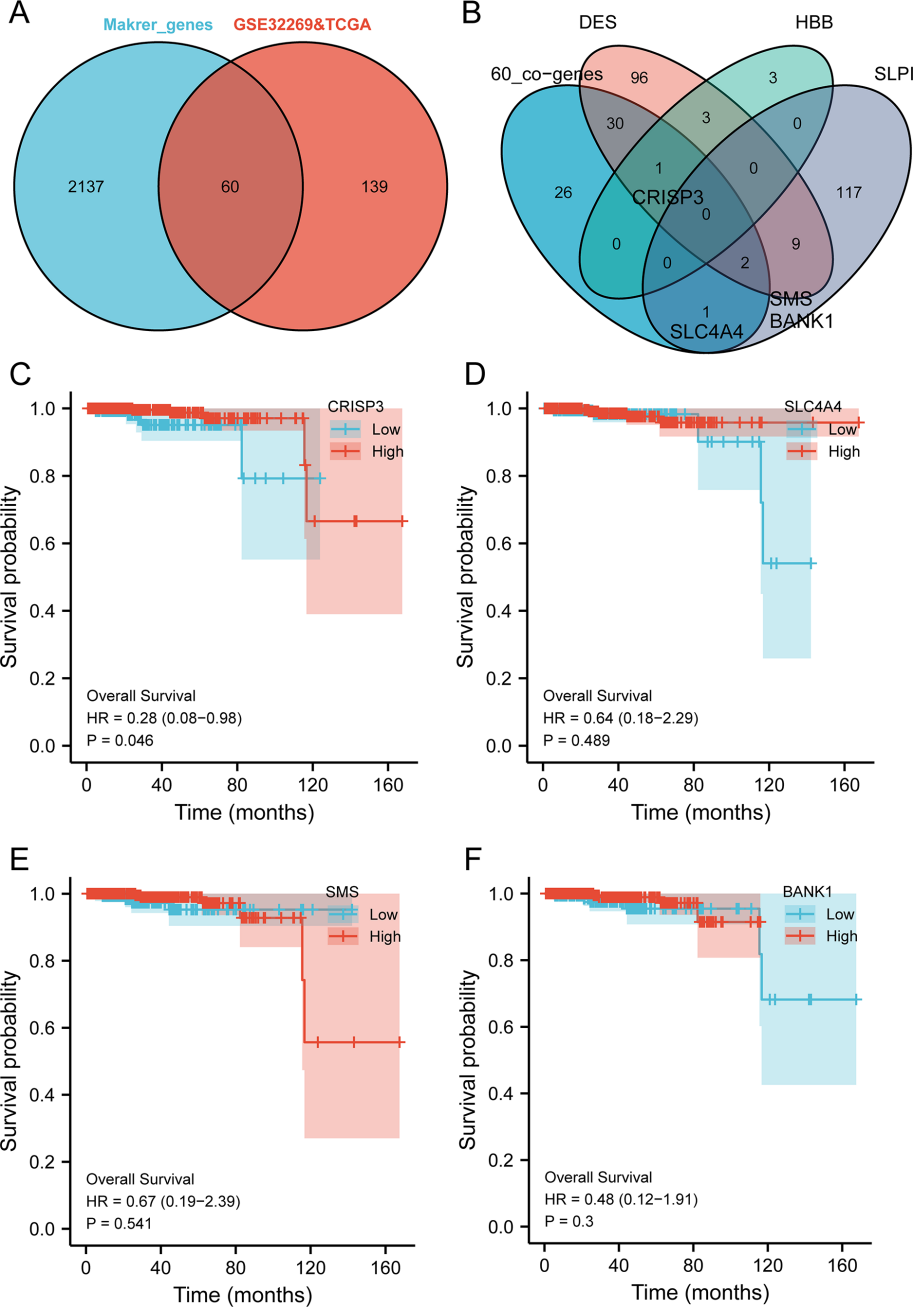
An examination of pivotal genes for bone metastatic prostate cancer (BMPCa). (A) Intersection analysis of the marker genes from single-cell GSE168733 with differential genes in BMPCa; (B) intersection analysis of 60 commonly differential genes with genes linked to expression of DES, HBB, and SLPI; and (C–F) Survival analysis of CRISP3 (C), SLC4A4 (D), SMS (E), and BANK1 (F) using the TCGA database.

The intersection analysis of marker genes of single-cell GSE168733 with differential genes for BMPCa revealed 60 key genes (Fig. 6A). An intersection analysis of these 60 common DEGs with genes associated with *DES*, *HBB*, and *SLPI* expression resulted in the identification of *CRISP3*, *SLC4A4*, *SMS*, and *BANK1* genes associated with BMPCa (Fig. 6B). Among these, only *CRISP3* expression was found to be associated with PCa prognosis (*p* < 0.05), suggesting that *CRISP3* may be a key gene for BMPCa associated with *DES* expression (Figs. 6C–6F). And CRISP3 was found as a key gene in BMPCa associated with DES expression. Volcano plot shows differential expression of DES, HBB, SLPI, and CRISP3 in prostate cancer bone metastases (Fig. S2).

### EMT is associated with CRISP3-induced proliferation and migration of bladder cancer cells

**Figure 7:**
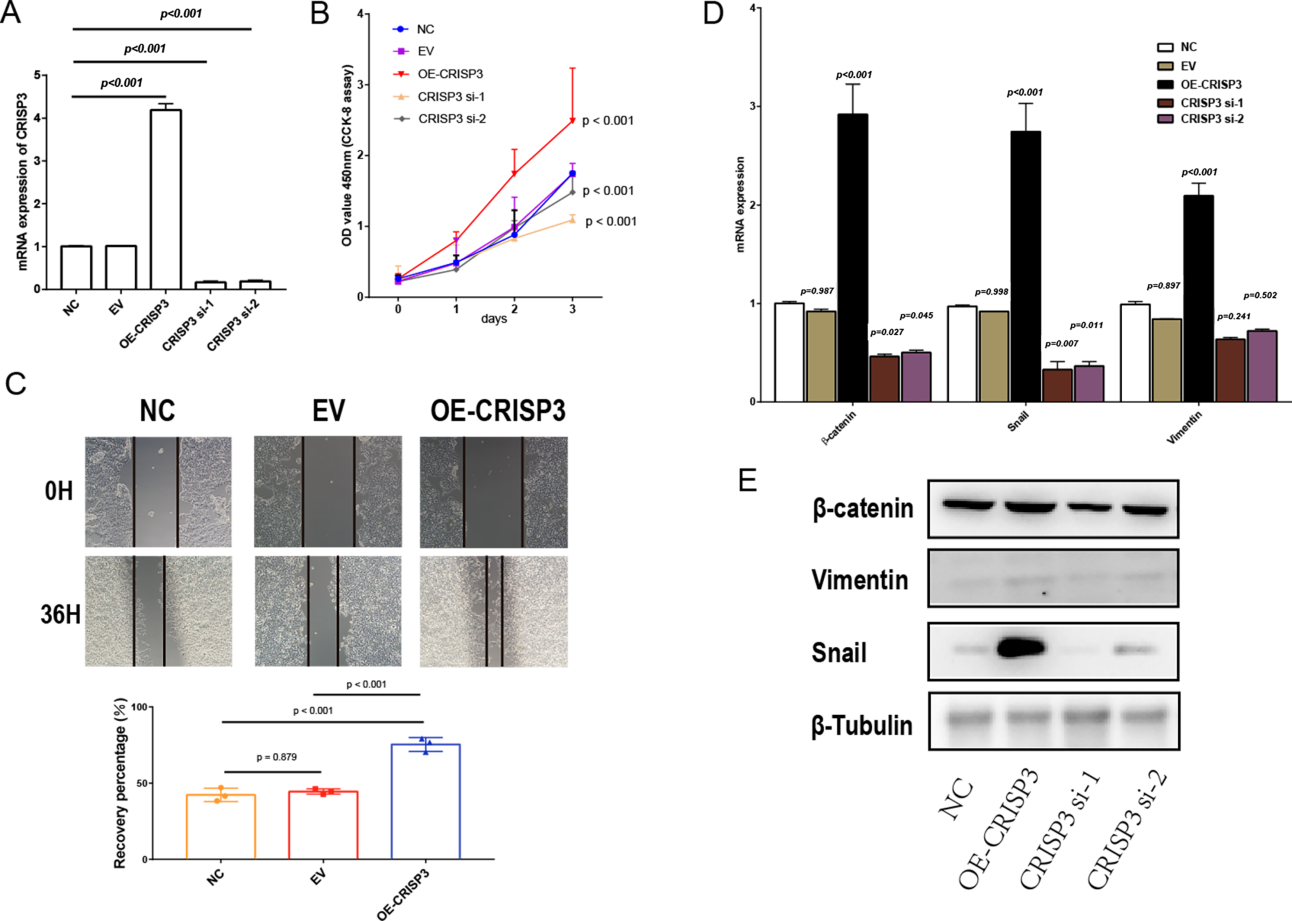
The proliferation and migration of bladder cancer cells elicited by CRISP3 is correlated with epithelial-to-mesenchymal transition (EMT). (A) qRT-PCR analysis of CRISP3 expression in prostate cancer cell lines; (B) results from the CCK8 assay suggest that the expression of CRISP3 significantly impacted cellular viability; (C) CRISP3 cells underwent transfection with negative control or empty vector or were overexpressed with CRISP3 after being treated with an invasion inhibitor; (D–E) qRT-PCR (D) and Western blot (E) were employed to assess the expression of EMT-related mRNA and proteins.

To further confirm that CRISP3 related to DES promotes prostate cancer proliferation, we carried out experiments in which CRISP3 was either overexpressed or silenced in LNCaP cells. Results obtained through qRT-PCR showed that the expression of CRISP3 was significantly reduced in the group treated with si-CRISP3 compared to the si-NC group. On the other hand, expression of CRISP3 was remarkably elevated in the group subjected to overexpression of the protein (Fig. 7A). The proliferation capacity and wound healing ability of LNCaP cells were remarkably enhanced upon overexpression of CRISP3, while the viability of CCK-8 cells was inhibited following silencing of CRISP3. No notable changes were observed in their respective negative controls (Figs. 7B–7C). It appears that CRISP3 may foster prostate cancer proliferation and migration by promoting EMT, as evidenced by the suppression of N-cadherin, Vimentin, and Snail markers of EMT after knocking down hsa_circ_0003823 protein (Figs. 7D–7E). Therefore, CRISP3 related to DES has been correlated with a poor prognosis in PCa, and it may induce tumor proliferation by promoting EMT.

## Discussion

Prostate cancer (PCa) is one of the most common malignancies in men worldwide and characterized by a high incidence of BM (21, 22). The majority of cancer-related deaths are caused by metastasis, yet this complex process is underpinned by the tumour microenvironment (Chambers, Groom & MacDonald, 2002; Lambert, Pattabiraman & Weinberg, 2017). Therefore, an in-depth understanding of the micro-environment immune mechanisms will contribute to the prevention and treatment of metastatic tumors.

Differential gene intersection and immune infiltration analysis revealed that *DES*, *HBB*, and *SLPI* were closely associated with immune cells. DES-associated CRISP3 is associated with poor prognosis in PCa, and it may promote tumor proliferation by promoting EMT. *DES* encodes desmin, a muscle-specific intermediate filament protein that plays a fundamental role in muscle structure and force transmission (Loreto et al., 2022). Mutations in *DES* result in abnormal cell growth patterns and adhesion, reduced RNA expression of *DES* and other membrane protein encoding genes, and desmin aggregation, causing abnormal cell function (Bermúdez-Jiménez et al., 2018). Additionally, desmin is involved in epithelial mesenchymal transition, promotes cell migration, and increases albumin flux in the cellular mono-molecular layer (Kang et al., 2010). These results are consistent with those obtained following enrichment of differential gene pathways. In this study, *DES* is investigated for the first time in relation to prostate cancer progression, and unlike previous studies, patients with high DES expression tend to have better outcomes. High *DES* expression is associated with the infiltration of resting CD4 memory T cells and naive B cells.

*HBB* gene encodes hemoglobin subunit beta, an important structural component constituting the hemoglobin tetramer. KM survival curves with low expression for HBB indicates poor tumour outcomes in this study. According to a related study, HBB may be a novel tumour suppressor gene in anaplastic thyroid cancer (ATC) (Onda et al., 2005). PCa was found to have high HBB variations, and it was also considered a potential prognostic biomarker (Davalieva et al., 2017; Lin et al., 2021). And high HBB expression may promote neutrophils infiltration. However, blocking the oxygen binding site of HBB reverses the increased tumor cell migration and upregulation of HIF-1 *α* in *HBB* over-expressing breast cancer cells (Ponzetti et al., 2017). HBB probably has different effects in different tumours, indicating that further pan-cancer research is necessary.

BMPCa patients are prone to fractures and bone pain due to activation of abnormal bone metabolic pathways. Secretory leukocyte peptidase inhibitor (SLPI) is a highly upregulated inhibitor of cellular proteases that protects HIF-2-alpha (2 *α*)-treated chondrocytes from inflammatory responses (Kim et al., 2021). In addition, SLPI active reticulum-like structures (a mixture of SLPI with neutrophil DNA and NE) stimulate the synthesis of type I interferon (IFNI) synthesis in plasmacytoid dendritic cells (pDCs) *in vitro* to regulate organismal immunity (Majewski et al., 2016). SLPI -treated monocyte culture supernatants inhibited the proliferation of CD4 ^+^ lymphocytes, but not of CD8 ^+^ cells, suggesting that SLPI can modulate innate and adaptive immune responses (Guerrieri et al., 2011).

CRISP3 was found to be a key gene in BMPCa associated with DES expression in this study. As a result of the transgenic mouse model of prostate cancer, CRISP3 production greatly contributed to the progression of *in situ* prostate cancer to invasive prostate cancer *in vivo* (Volpert et al., 2020). PCa containing the TMPRSS2-ERG fusion gene overexpresses CRISP3, a direct target of ERG. There is also some evidence that CRISP-3 may be associated with the development of pancreatic cancer lesions in other types of tumours, particularly those which are predominantly found in the gastrointestinal tract (Liao et al., 2003). CRISP3 has been identified as a potential therapeutic target in PCa progression (Noh et al., 2016; Volpert et al., 2020).

D- Glucopyranose is a synonym for D-glucose, one of the most common glucose isomers in nature, and plays an important role in glucolipid metabolism. There is evidence that the acquired metabolic phenotype associated with androgen receptor (AR)-targeted therapies is related to glucose and lipid metabolism disorders (Blomme et al., 2020). Additionally, high levels of serum glucose and triglycerides may affect PCa severity and aggressiveness (Arthur et al., 2016). According to another study, elevated levels of glycolipid metabolism biomarkers before prostate cancer diagnosis were associated with an increased risk of secondarily diagnosed primary tumours (Bosco et al., 2018). This study indicates that DES-associated CRISP3 is associated with poor prognosis in PCa, and it may promote tumor proliferation and metastatic capacity by promoting EMT.

A preliminary bioinformatics analysis of the results in this study demonstrated that *CRISP3*, *DES*, *HBB*, and *SLPI* in the bone micro-environment are capable of inhibiting BM by regulating lipid metabolism and immune cell infiltration. However, most of the existing studies are based on bioinformatics data analysis, and the results of this study need to be further validated by *in vitro* and *in vivo* studies to develop a better understanding of the immune mechanisms and prognosis of BMPCa.

## Conclusion

*DES*, *HBB*, and *SLPI* may inhibit the development of bone metastasis in patients with PCa by regulating immune cell infiltration. Cellular experiments revealed that DES-associated CRISP3 is associated with poor prognosis in PCa, and it may promote tumor proliferation and metastatic capacity by promoting EMT.

##  Supplemental Information

10.7717/peerj.15013/supp-1 Supplemental Information 1 Sequences of CRISP3 siRNA used in this study

10.7717/peerj.15013/supp-2 Supplemental Information 2 Sequences of primers used for qPCR in this study

10.7717/peerj.15013/supp-3 Supplemental Information 3 Raw data and plot

10.7717/peerj.15013/supp-4 Supplemental Information 4 The top 20 genes are ranked *via* the Random Forest Model

10.7717/peerj.15013/supp-5 Supplemental Information 5 A volcano plot illustrates the differential expression of DES, HBB, SLPI, and CRISP3 in bone metastases of prostate cancer

## Additional Information and Declarations

### Data Availability

The following information was supplied regarding data availability: The raw measurements are available in the Supplemental Files.
