# Supplementary material for: Exploring the role of differentially expressed metabolic genes and their mechanisms in bone metastatic prostate cancer
Source: PeerJ. 2023 Apr 12;11:e15013. doi: 10.7717/peerj.15013 (PMC10105558; doi:10.7717/peerj.15013)
Supplement: Supplemental Information 1 [file peerj-11-15013-s001.docx]

| Table S1 Sequences of CRISP3 siRNA used in this study | | |
| --- | --- | --- |
| CRISP3 si-1 | sense | 5'-AGUAACCCAAAGGAUCGAATT-3' |
| CRISP3 si-1 | antisense | 5'-UUCGAUCCUUUGGGUUACUTT-3' |
| CRISP3 si-2 | sense | 5'-GUUGGAUGUGGAAAUGCCUTT-3' |
| CRISP3 si-2 | antisense | 5'-AGGCAUUUCCACAUCCAACTT-3' |
| CRISP3 NC | sense | 5'-UUCUCCGAACGUGUCACGUdTdT-3' |
| CRISP3 NC | antisense | 5'-ACGUGACACGUUCGGAGAAdTdT-3' |
