# Supplementary material for: Exploring the role of differentially expressed metabolic genes and their mechanisms in bone metastatic prostate cancer
Source: PeerJ. 2023 Apr 12;11:e15013. doi: 10.7717/peerj.15013 (PMC10105558; doi:10.7717/peerj.15013)
Supplement: Supplemental Information 2 [file peerj-11-15013-s002.docx]

| Table S2 Sequences of primers used for qPCR in this study | |
| --- | --- |
| CRISP3-F | ACCTCGTTGGATGTGGAAAT |
| CRISP3-R | CAACCATTGGTGCATAGTCC |
| GAPDH-F | GAAGGTGAAGGTCGGAGTC |
| GAPDH-R | GAAGATGGTGATGGGATTTC |
