# Supplementary figures and images for: Exploring the role of differentially expressed metabolic genes and their mechanisms in bone metastatic prostate cancer
Source: PeerJ. 2023 Apr 12;11:e15013. doi: 10.7717/peerj.15013 (PMC10105558; doi:10.7717/peerj.15013)

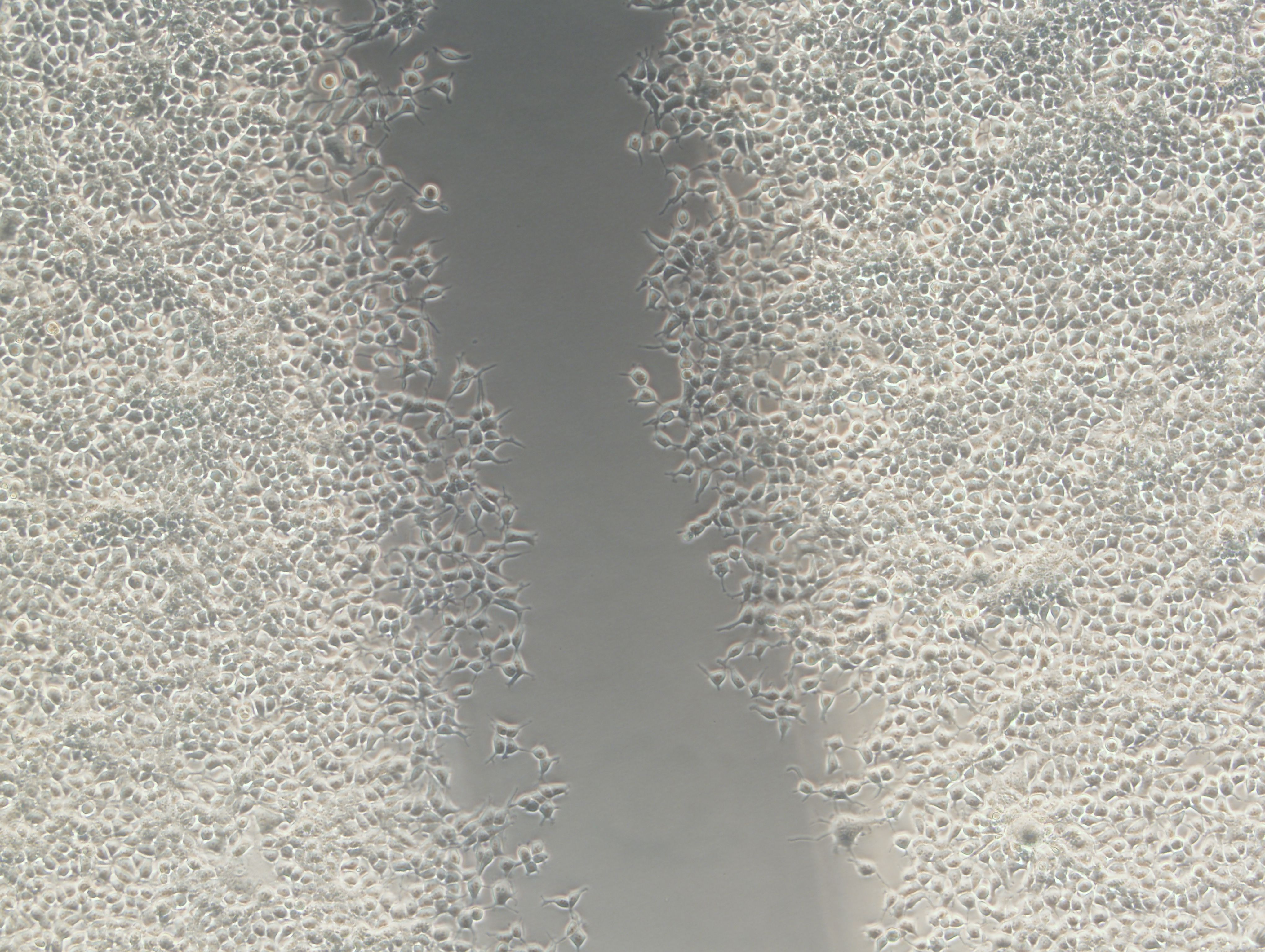

Supplement: Supplemental Information 3 [file peerj-11-15013-s003.zip › rawdata and plot/scratch assay/EC-H.jpg]

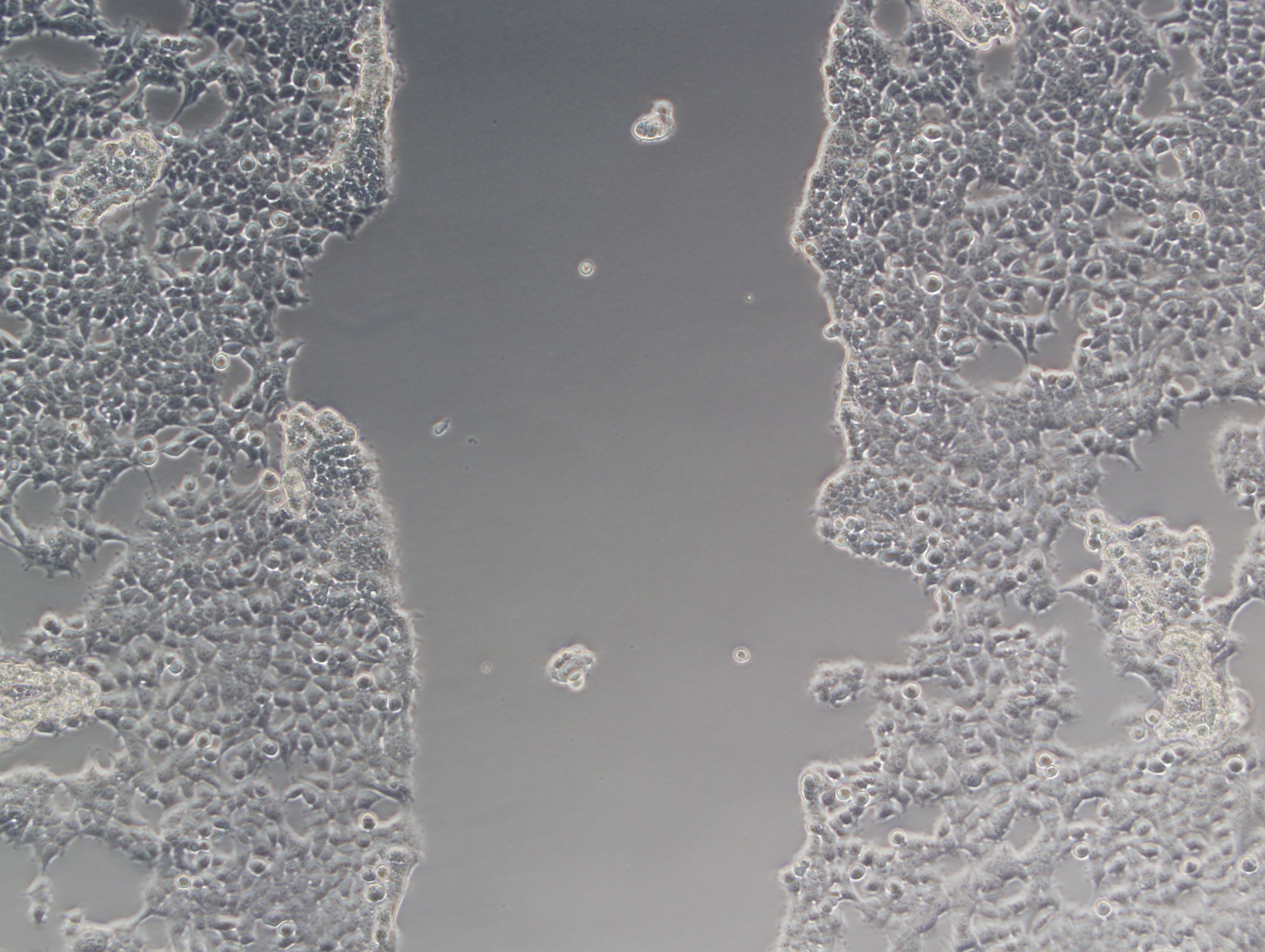

Supplement: Supplemental Information 3 [file peerj-11-15013-s003.zip › rawdata and plot/scratch assay/EC.jpg]

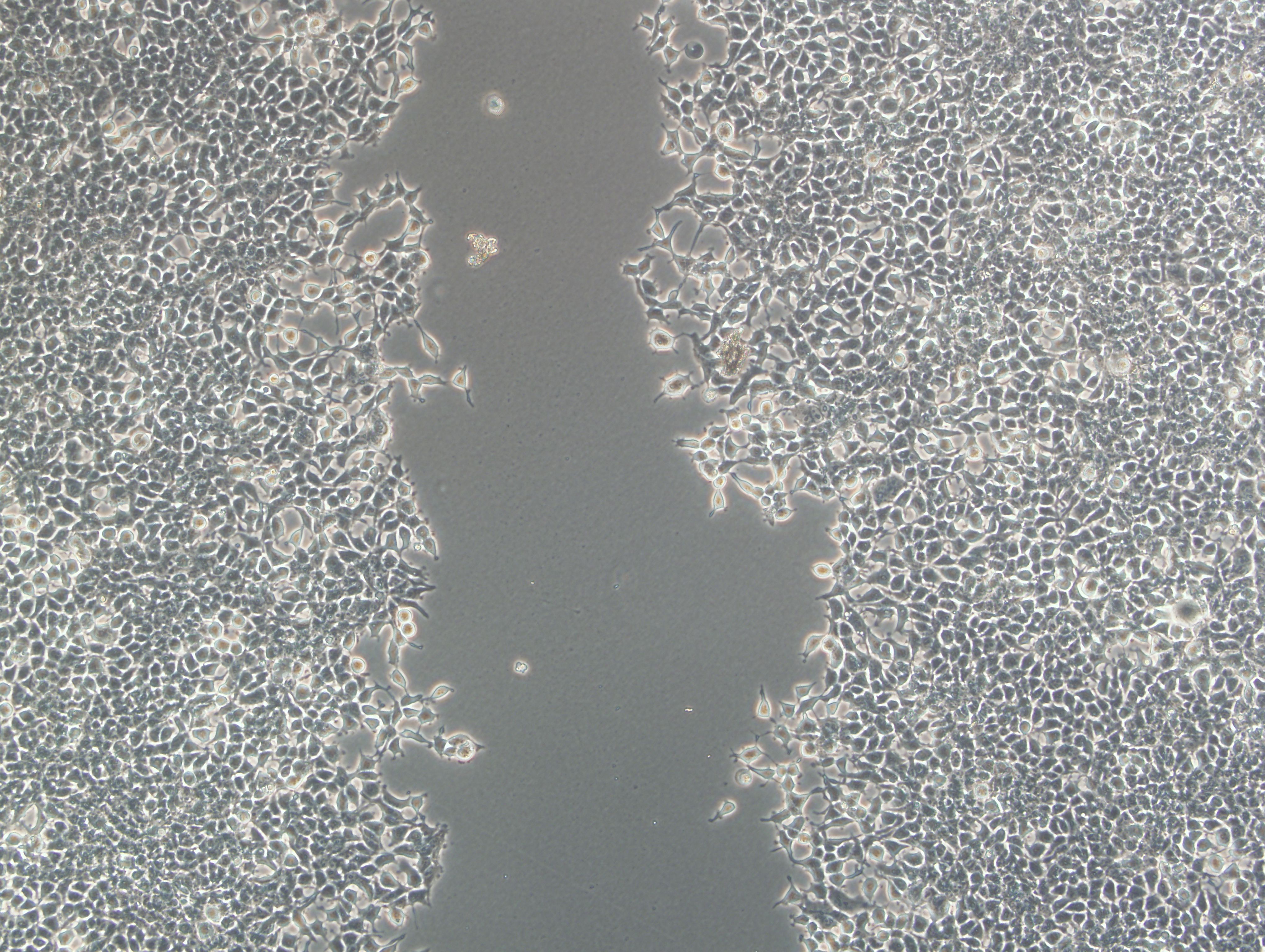

Supplement: Supplemental Information 3 [file peerj-11-15013-s003.zip › rawdata and plot/scratch assay/NC-H.jpg]

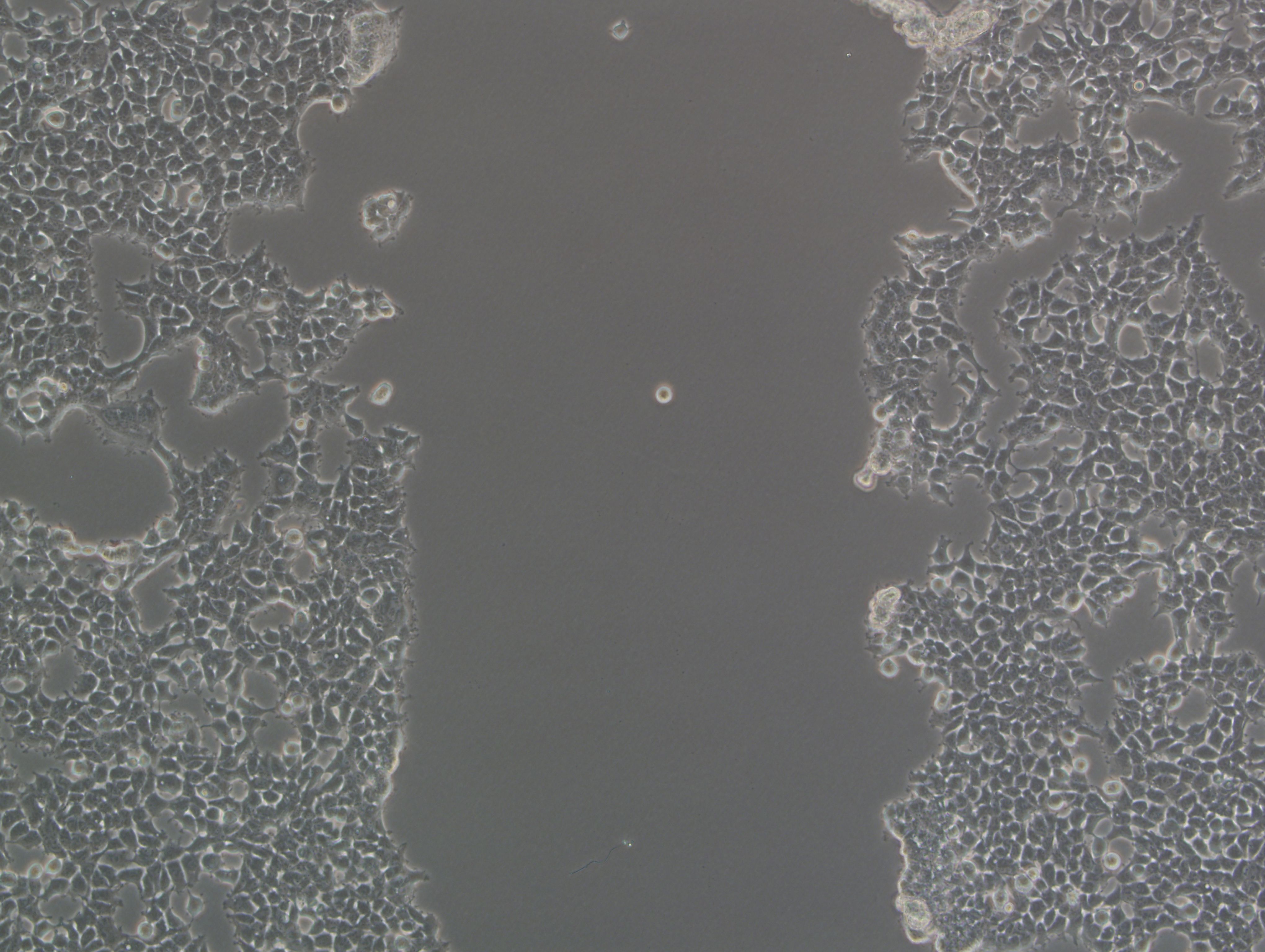

Supplement: Supplemental Information 3 [file peerj-11-15013-s003.zip › rawdata and plot/scratch assay/NC.jpg]

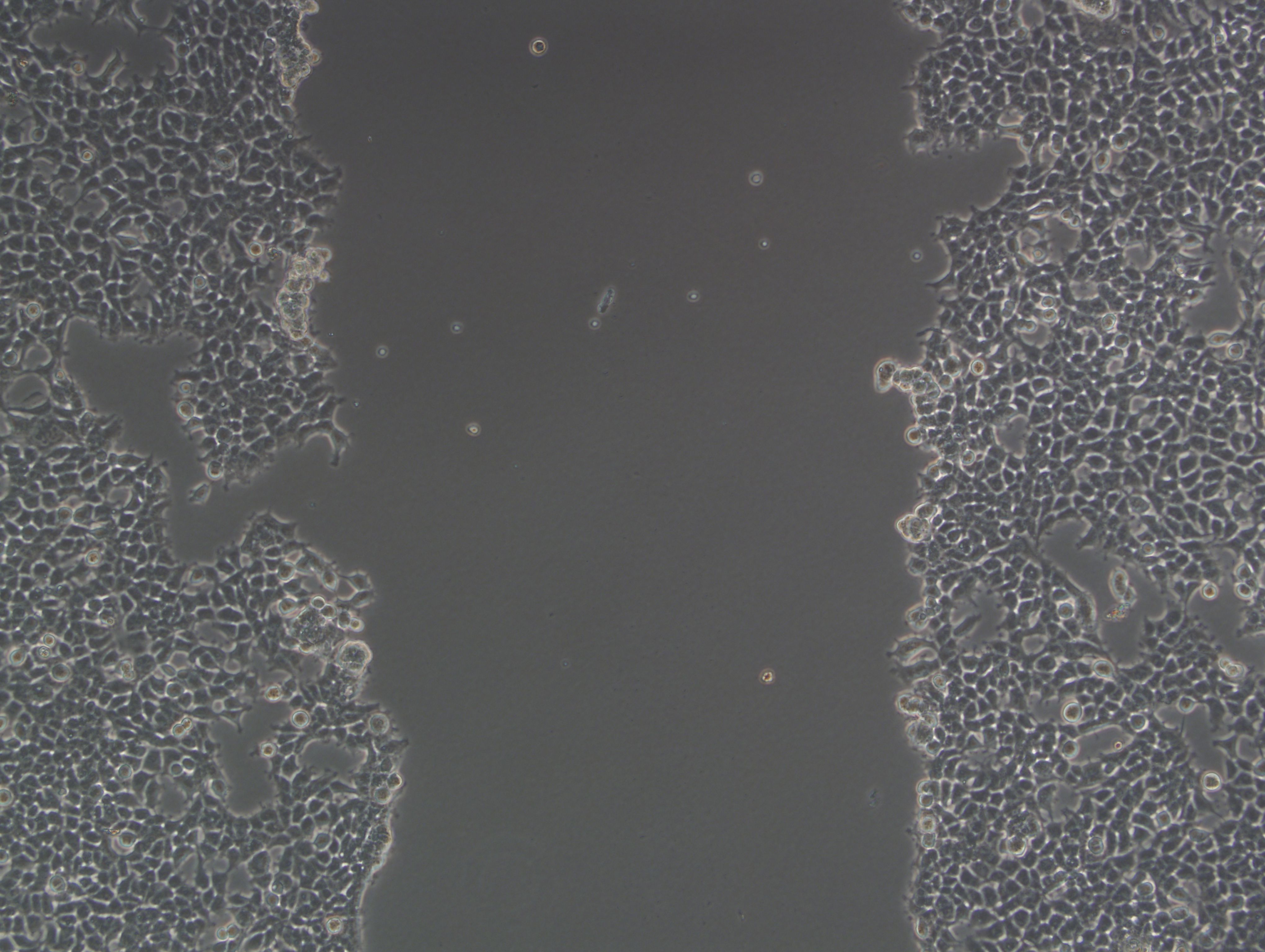

Supplement: Supplemental Information 3 [file peerj-11-15013-s003.zip › rawdata and plot/scratch assay/OE-CRISP3.jpg]

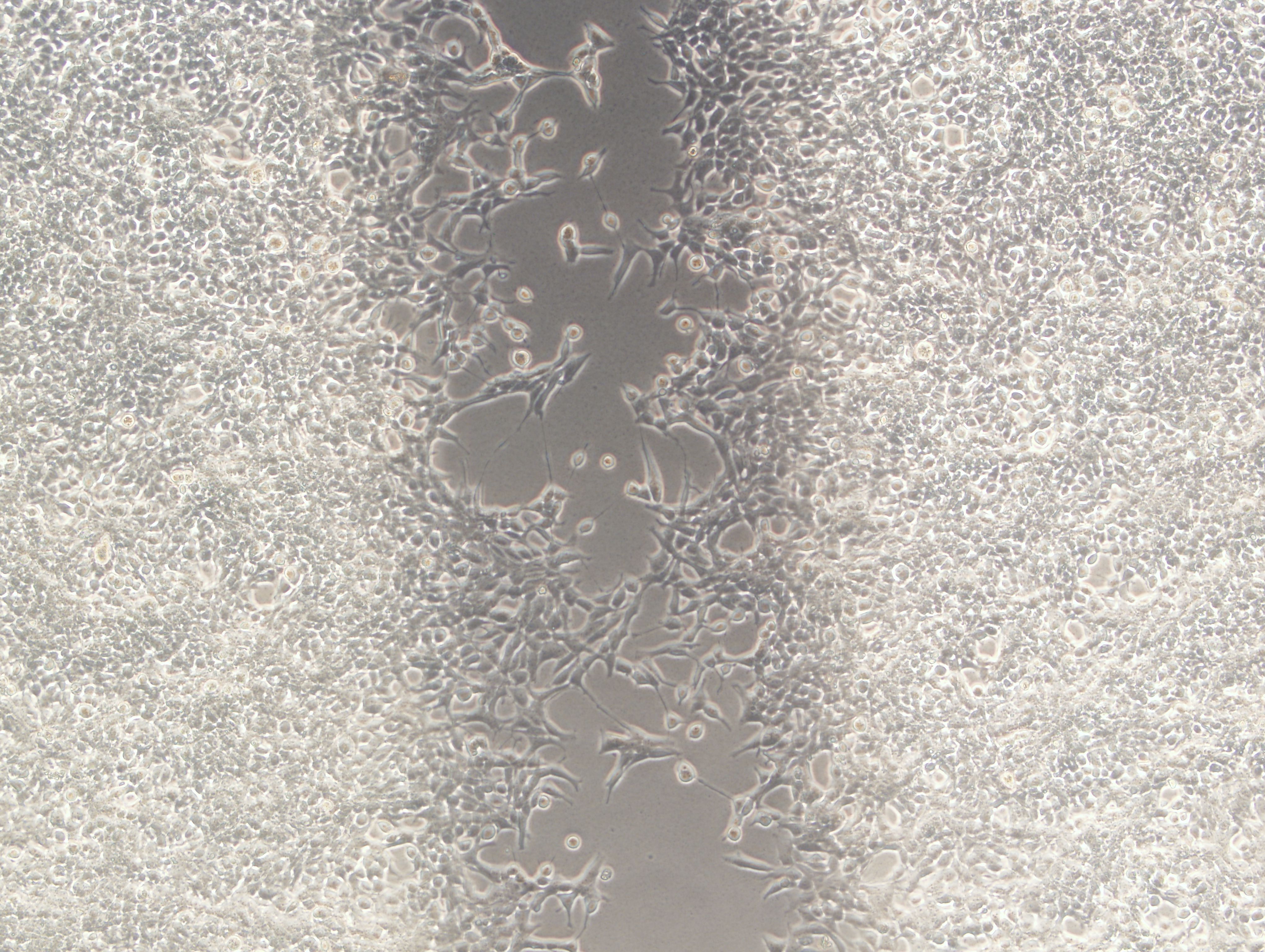

Supplement: Supplemental Information 3 [file peerj-11-15013-s003.zip › rawdata and plot/scratch assay/OE-CRISP3H.jpg]

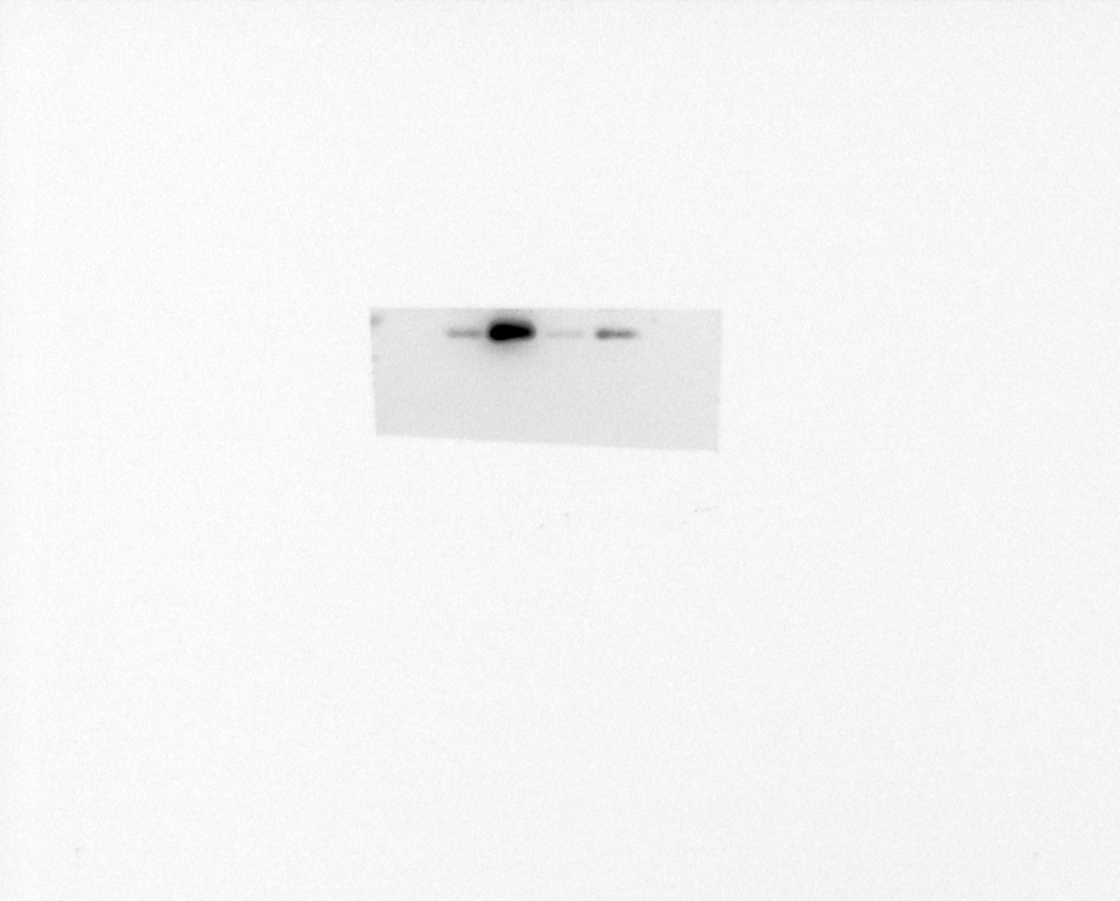

Supplement: Supplemental Information 3 [file peerj-11-15013-s003.zip › rawdata and plot/WB/Snail 29kda/Snail 29kda (2).tif]

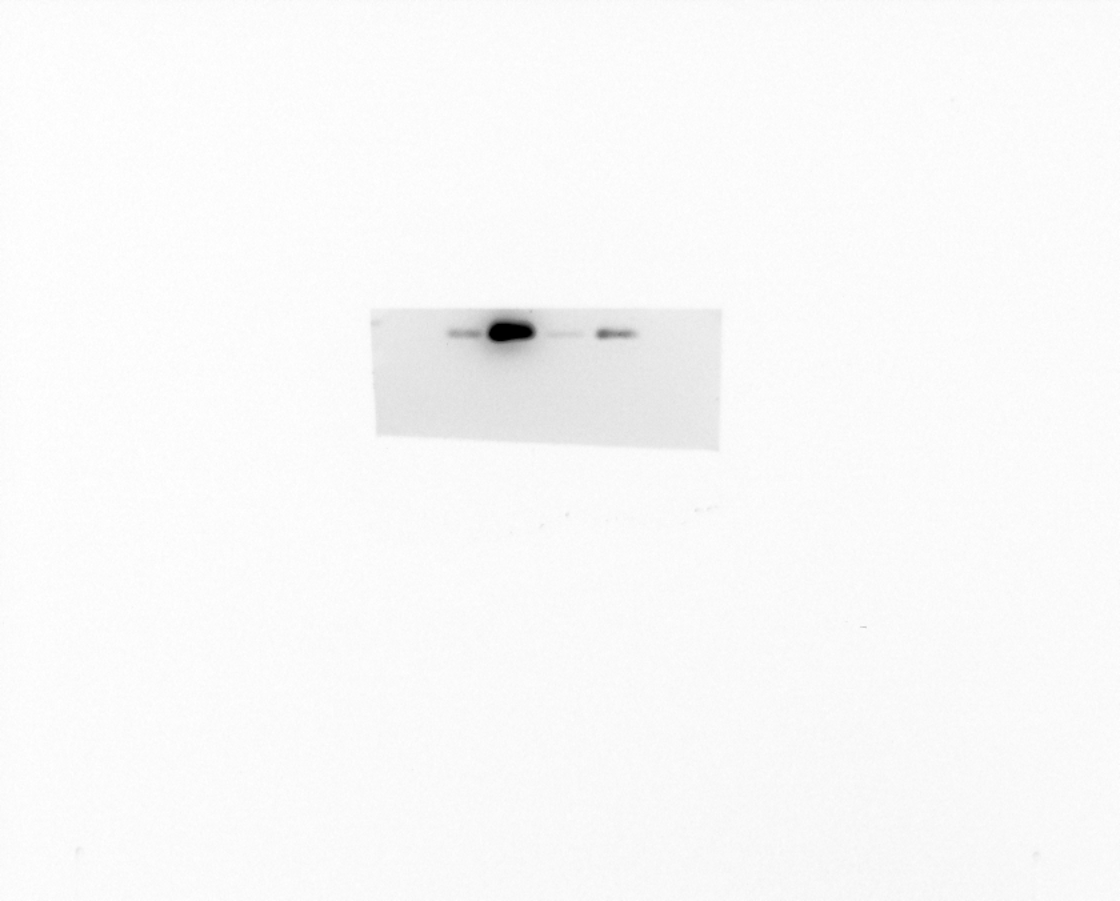

Supplement: Supplemental Information 3 [file peerj-11-15013-s003.zip › rawdata and plot/WB/Snail 29kda/Snail 29kda (3).tif]

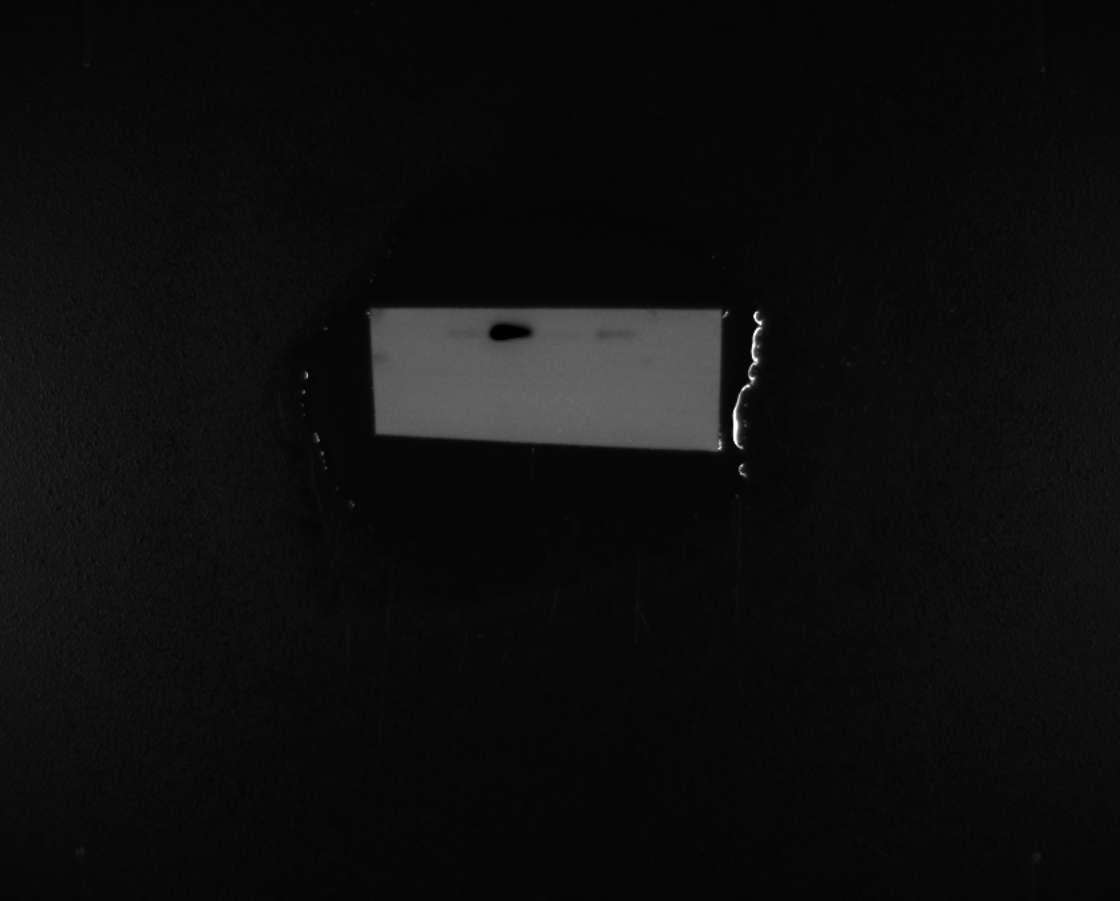

Supplement: Supplemental Information 3 [file peerj-11-15013-s003.zip › rawdata and plot/WB/Snail 29kda/Snail 29kda.tif]

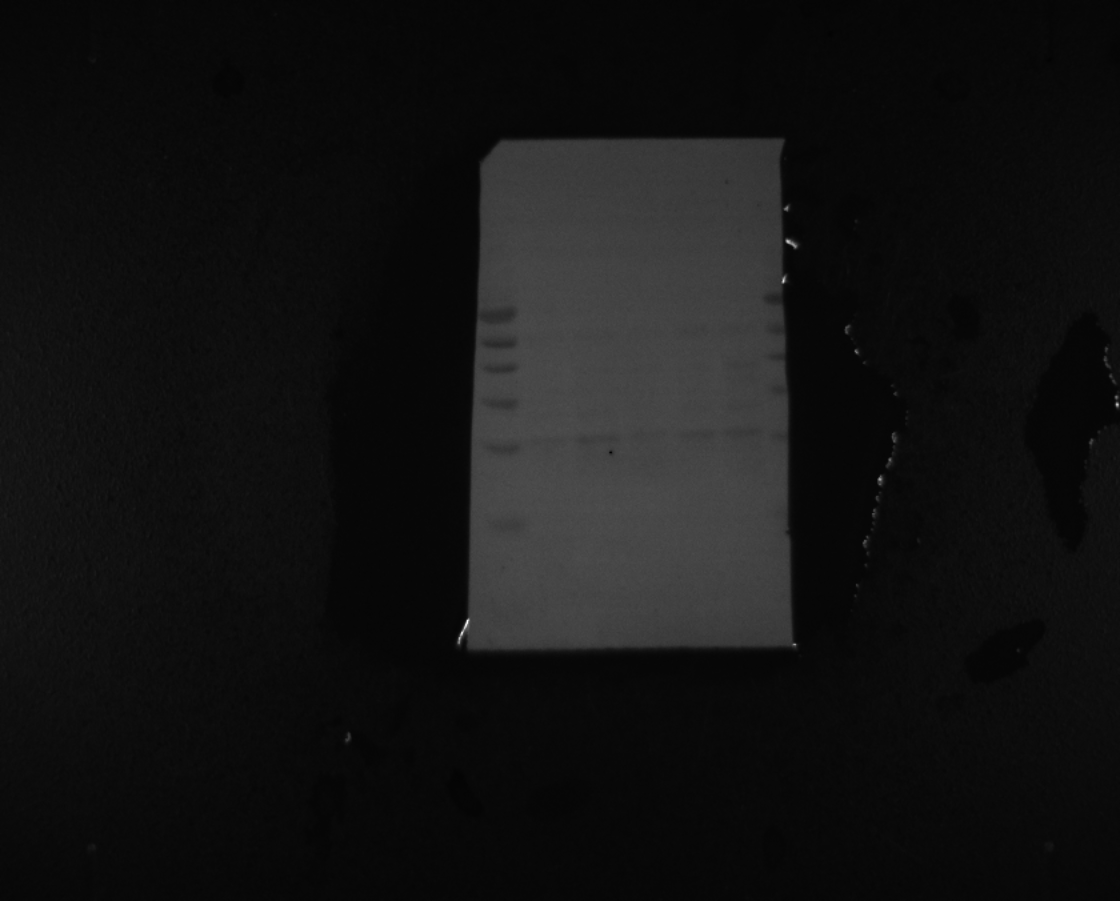

Supplement: Supplemental Information 3 [file peerj-11-15013-s003.zip › rawdata and plot/WB/Vimentin 55kda/Vimentin 55kda (2).tif]

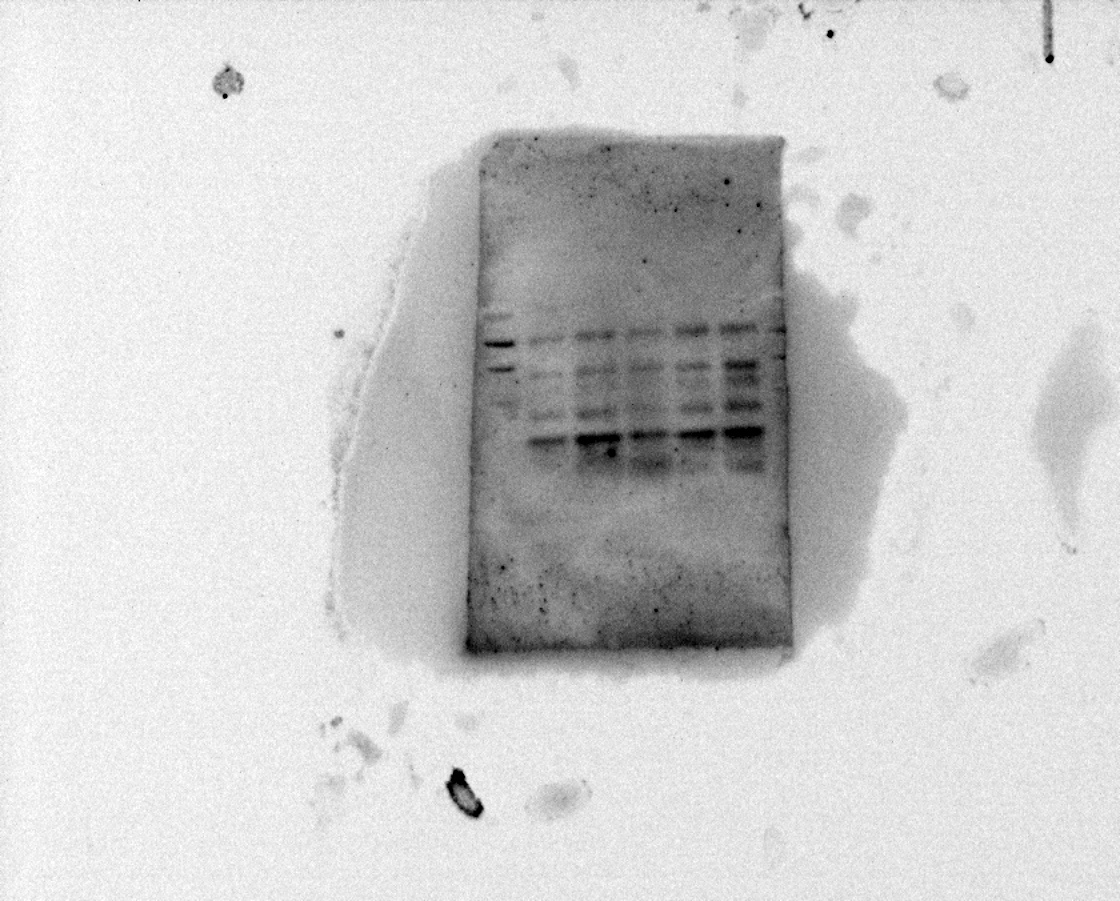

Supplement: Supplemental Information 3 [file peerj-11-15013-s003.zip › rawdata and plot/WB/Vimentin 55kda/Vimentin 55kda.tif]

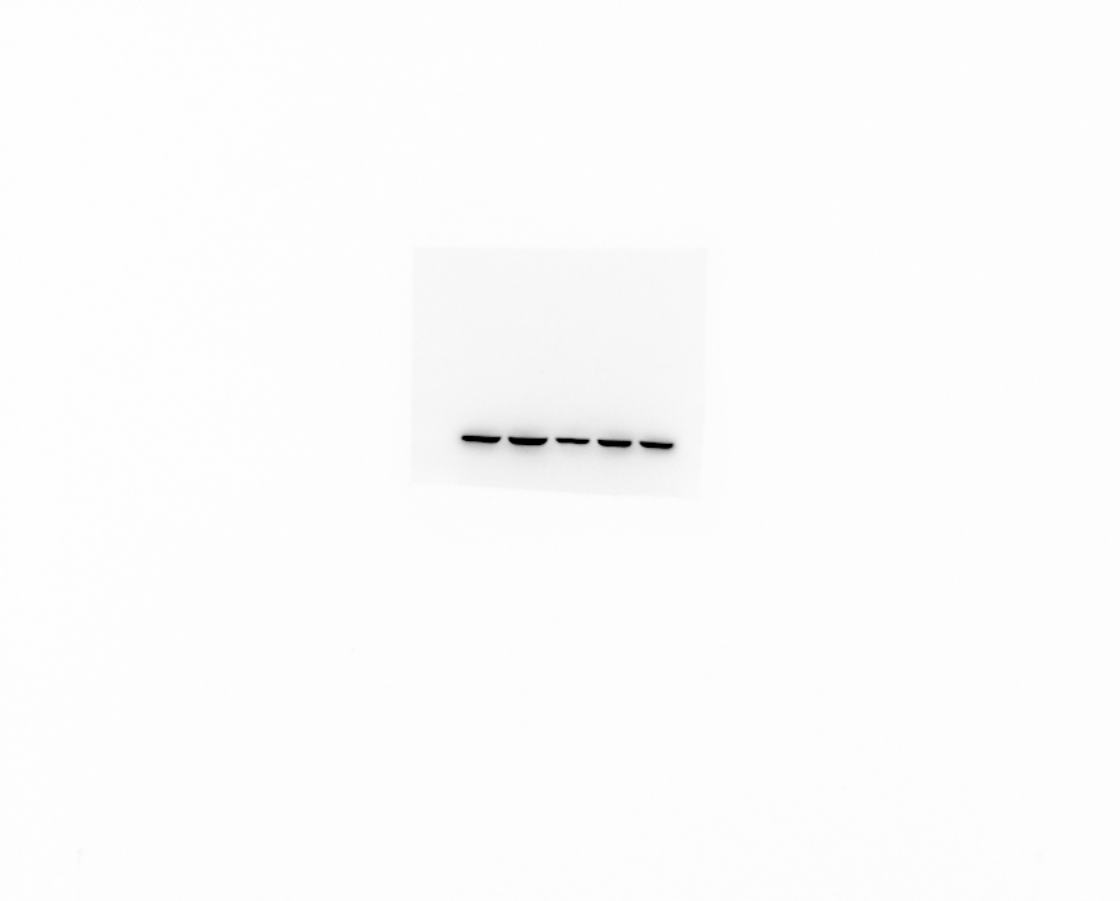

Supplement: Supplemental Information 3 [file peerj-11-15013-s003.zip › rawdata and plot/WB/β-catenin 95kda/β-catenin 95kda (3).tif]

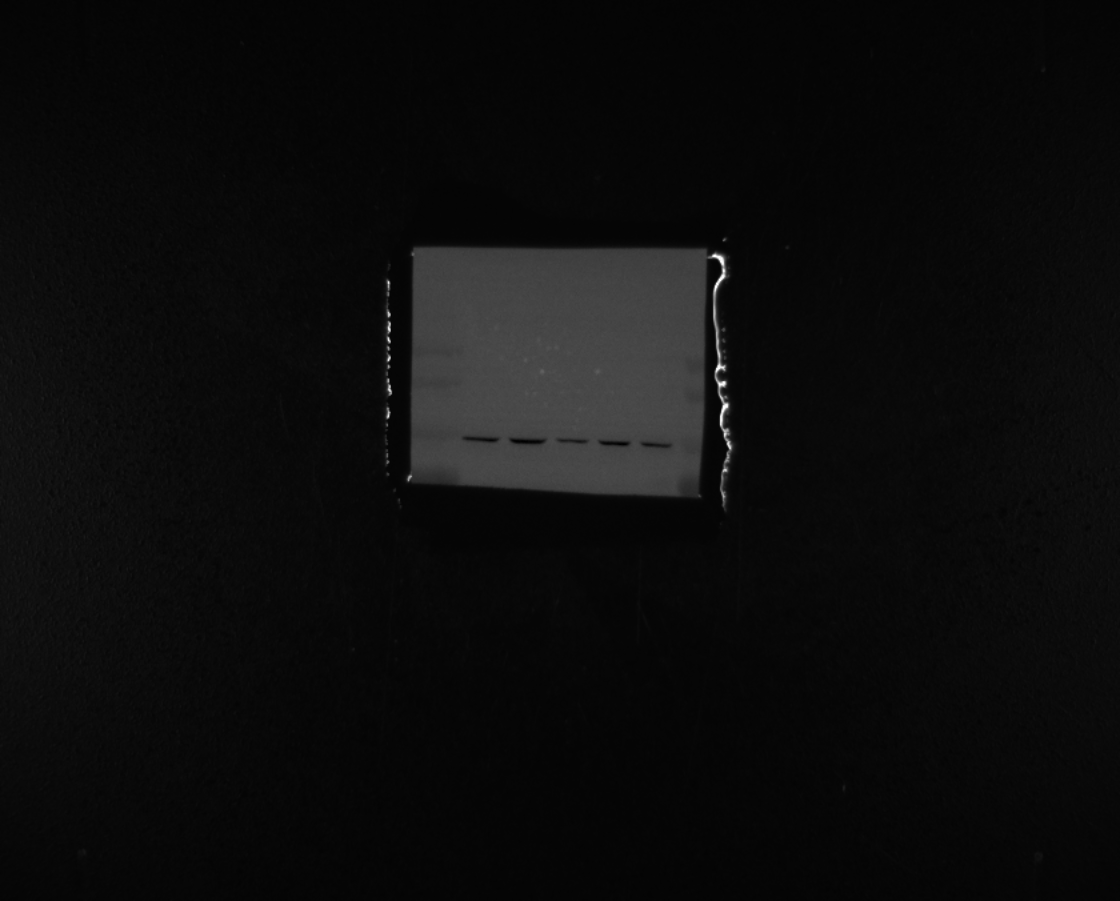

Supplement: Supplemental Information 3 [file peerj-11-15013-s003.zip › rawdata and plot/WB/β-catenin 95kda/β-catenin 95kda.tif]

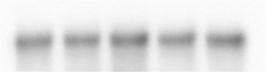

Supplement: Supplemental Information 3 [file peerj-11-15013-s003.zip › rawdata and plot/WB/β-Tubulin 55kda/β-Tubulin_400ms (2).png]

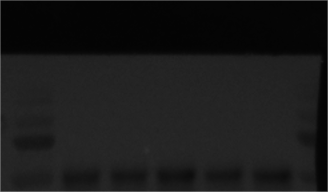

Supplement: Supplemental Information 3 [file peerj-11-15013-s003.zip › rawdata and plot/WB/β-Tubulin 55kda/β-Tubulin_400ms.png]

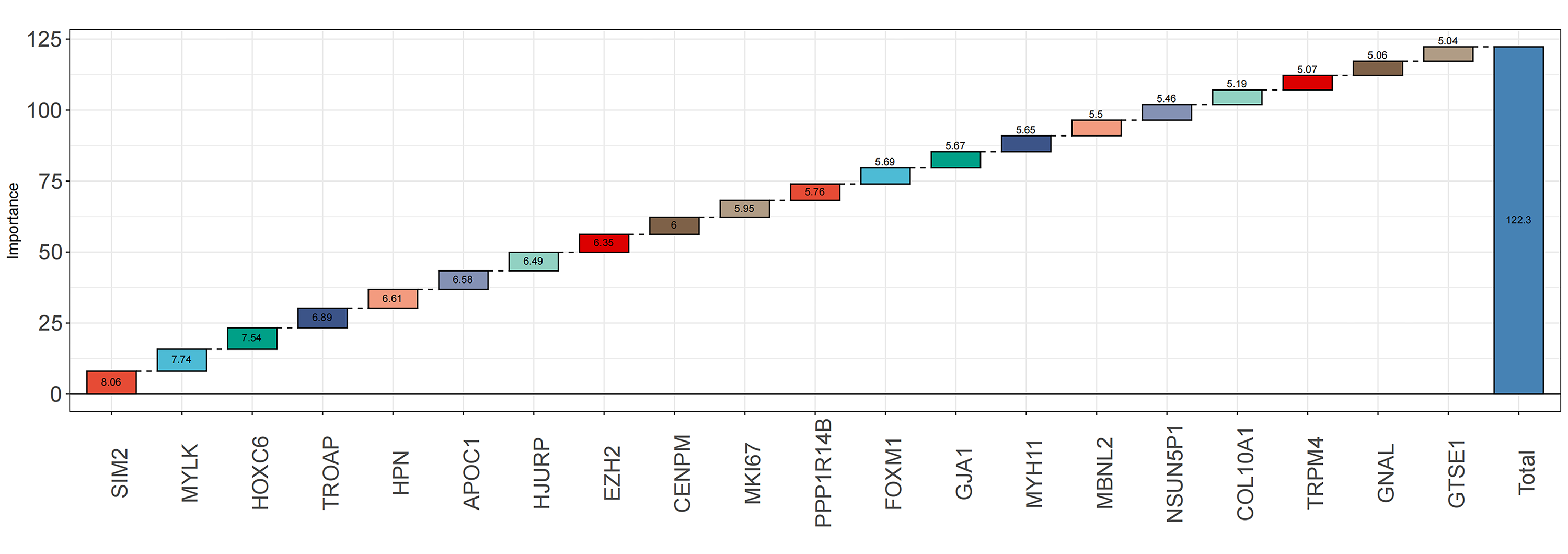

Supplement: Supplemental Information 4 [file peerj-11-15013-s004.png]

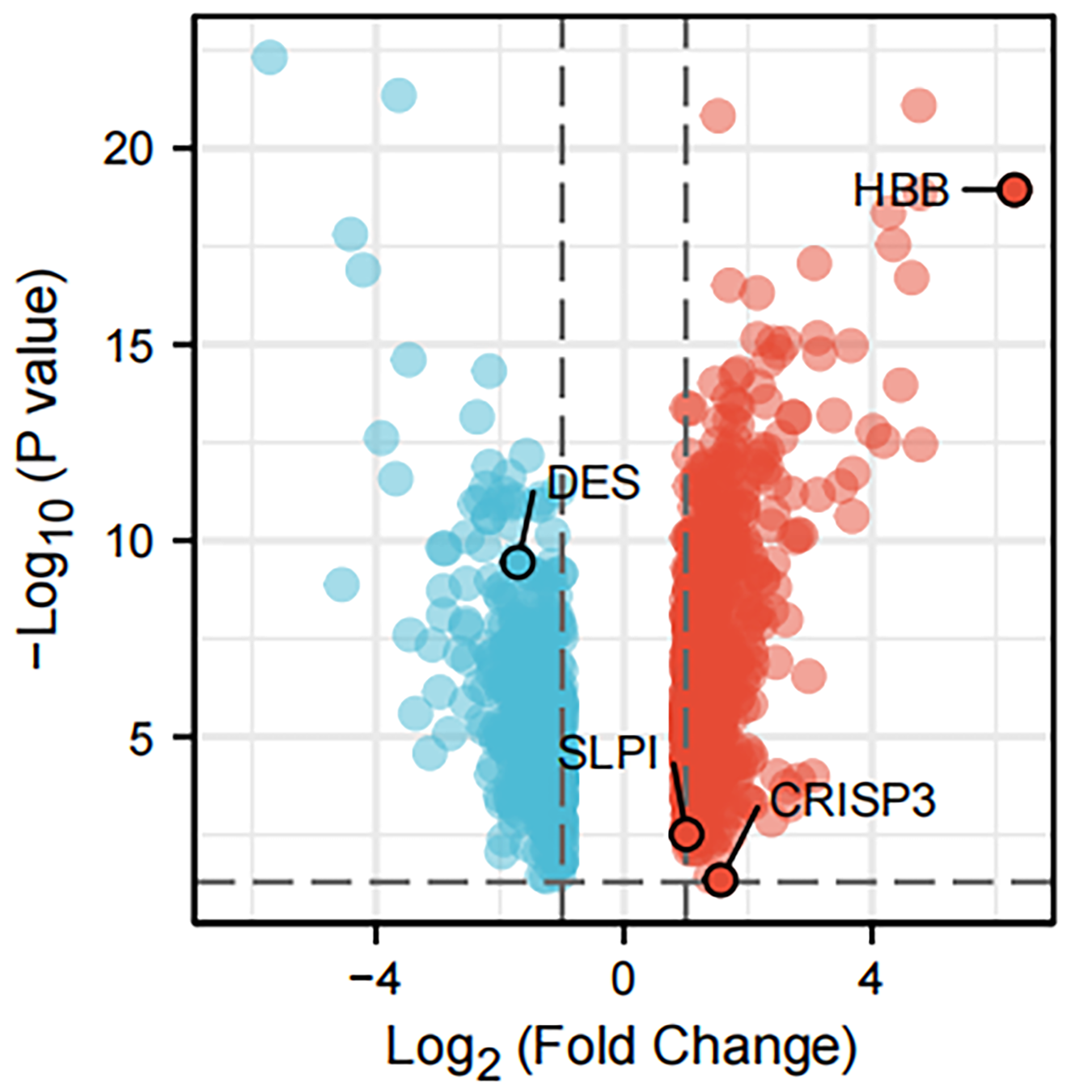

Supplement: Supplemental Information 5 [file peerj-11-15013-s005.png]
